# CRISPR/Cas9‐mediated PBP1 and PBP3 mutagenesis induced significant reduction in electrophysiological response to sex pheromones in male *Chilo suppressalis*

**DOI:** 10.1111/1744-7917.12544

**Published:** 2017-12-07

**Authors:** Xiao‐Tong Dong, Hui Liao, Guan‐Heng Zhu, Sajjad Ali Khuhro, Zhan‐Feng Ye, Qi Yan, Shuang‐Lin Dong

**Affiliations:** ^1^ College of Plant Protection Nanjing Agricultural University/Key Laboratory of Integrated Management of Crop Diseases and Pests Ministry of Education Nanjing China

**Keywords:** CRISPR/Cas9 system, electroantennogram, pheromone binding protein, sex pheromone, striped stem borer

## Abstract

Pheromone‐binding proteins (PBPs) are thought to bind and transport sex pheromones onto the olfactory receptors on the dendrite membrane of olfactory neurons, and thus play a vital role in sex pheromone perception. However, the function of PBPs has rarely been demonstrated *in vivo*. In this study, two PBPs (PBP1 and PBP3) of *Chilo suppressalis*, one of the most notorious pyralid pests, were *in vivo* functionally characterized using insects with the PBP gene knocked out by the CRISPR/Cas9 system. First, through direct injection of PBP‐single guide RNA (sgRNA)/Cas9 messenger RNA into newly laid eggs, a high rate of target‐gene editing (checked with polled eggs) was induced at 24 h after injection, 21.3% for PBP1‐sgRNA injected eggs and 19.5% for PBP3‐sgRNA injected eggs. Second, by an in‐crossing strategy, insects with mutant PBP1 or PBP3 (both with a premature stop codon) were screened, and homozygous mutants were obtained in the G3 generation. Third, the mutant insects were measured for electroantennogram (EAG) response to female sex pheromones. As a result, both PBP mutant males displayed significant reduction in EAG response, and this reduction in PBP1 mutants was higher than that in PBP3 mutants, indicating a more important role of PBP1. Finally, the relative importance of two PBPs and the possible off target effect induced by sgRNA‐injection are discussed. Taken together, our study provides a deeper insight into the function of and interaction between different PBP genes in sex pheromone perception of *C. suppressalis*, as well as a valuable reference in methodology for gene functional study in other genes and other moth species.

## Introduction

Moths have evolved a highly sophisticated and sensitive olfactory system to guide various important behaviors, such as locating food sources and finding mating partners. The major proteins involved in the olfactory system at the peripheral level includes odorant‐binding proteins (OBPs), odorant receptors (ORs), ionotropic receptors (IRs), sensory neuron membrane proteins (SNMPs) and odorant‐degrading enzymes (ODEs) (Leal, 2013). Among these proteins, OBP is a large family of small (∼15 kDa) hydrophilic carrier proteins that contain two‐four disulphide bridges. OBPs primarily function as solubilizers and carriers of the hydrophobic odorants in the aqueous sensillum lymph, thus protecting and transporting hydrophobic odorants to ORs on the dendritic membrane (Pelosi & Maida, 1995; Leal, 2005). As a sub‐class of OBPs in lepidopteran insects, pheromone binding proteins (PBPs), as the name designates, are responsible for transporting sex pheromones, although function in transporting plant volatiles has also been suggested (Liu *et al*., 2012a; Rouyar *et al*., 2015). In addition, PBPs have been indicated to serve as cofactors in the activation of PRs (Sun *et al*., 2013; Chang *et al*., 2015) and be involved in the postulated odorant molecule deactivation (Ziegelberger, 1995). Furthermore, multiple PBPs have been found in many moth species (Maida *et al*., 2000; Xiu & Dong, 2007; Cao *et al*., 2014). The existence of multiple PBPs in a species suggests that PBPs may differentiate in pheromone perception (Robertson *et al*., 1999; Picimbon & Gadenne, 2002), in terms of particular selectivity among different pheromone components and the relative importance in pheromone perception. However, the functions of PBPs have rarely been demonstrated by *in vivo* evidence, due to the lacking of suitable functional study technique. In particular, the commonly used *in vivo* technique of RNA interference (RNAi) is not effective or stable for the lepidopteran species (Terenius *et al*., 2011).

Very recently, the CRISPR/Cas9 system has provided us with a new and feasible approach for *in vivo* gene functional analysis in a fast and cheap way (Yue *et al*., 2016), and has been expanded in *Bombyx mori* and several other lepidopteran species. In these lepidopteran insects, genes related to embryogenesis (Liu *et al*., 2016), pigmentation (Wang *et al*., 2013b; Khan *et al*., 2017), metamorphosis (Li *et al*., 2015; Huang *et al*., 2016), physiological clock (Markert *et al*., 2016), and olfaction (Koutroumpa *et al*., 2016; Zhu *et al*., 2016) were studied by using the CRISPR/Cas9 technique. These studies demonstrated that the CRISPR/Cas9 system is highly efficient in inducing gene mutagenesis, and thus is a suitable *in vivo* technique for gene function study in both model and non‐model lepidopteran species.

The striped stem borer, *Chilo suppressalis*, is one of the most notorious rice pests in temperate regions of Asia. Due to the wide adoption of hybrid rice varieties, *C. suppressalis* is particularly damaging in China (Lou *et al*., 2013). The sex pheromone of female *C. suppressalis* has been identified as a blend of three components, including (*Z*)‐11‐hexadecenal (*Z*11‐16:Ald), (*Z*)‐13‐octadecenal (*Z*13‐18:Ald) and (*Z*)‐9‐hexadecenal (*Z*9‐16:Ald), with a ratio of 48:6:5 in the pheromone gland extract (Tatsuki, 1990). So far, four *C. suppressalis* PBP genes have been identified through transcriptomic analysis (Cao *et al*., 2014). The *in vitro* ligand binding assay showed that CsupPBP1 and CsupPBP3 had much higher affinities to all three components than PBP2 and PBP4 (Chang *et al*., 2015), suggesting that PBP1 and PBP3 play important roles in sex pheromone detection in *C. suppressalis*.

In the present study, we report a successful use of the CRISPR/Cas9 system in PBP functional study in the pyralid moth pest, *C. suppressalis*. High rates of mutagenesis targeting CsupPBP1 and CsupPBP3 were obtained by direct egg injection of PBP‐sgRNA/Cas9‐mRNA, and subsequent *in vivo* functional analysis was conducted by using the mutant insects. Our study provides direct evidence for importance of PBP1 and PBP3 in sex pheromone perception of *C. suppressalis*, as well as a valuable reference in methodology for gene functional study in other genes and in other moth species.

## Materials and methods

### Insects rearing

The striped rice stem borer *C. suppressalis* were reared in an insectarium at 28 ± 1°C, 90% ± 5% relative humidity, and photoperiod of 16 : 8 h (light : dark), with artificial diet (Han *et al*., 2012). Pupae were sexed before eclosion and kept in cages.

### Single guide RNA (sgRNA) design and in vitro synthesis of sgRNA and Cas9‐messenger RNA (mRNA)

**Figure 1:**
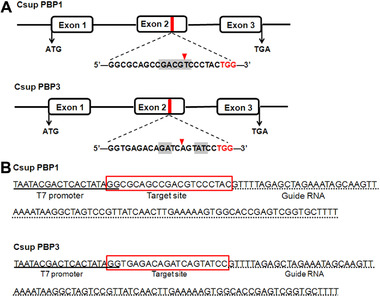
Diagram of target sites and elements of single guide RNA (sgRNA) of pheromone‐binding protein (PBP1) and PBP3 genes. (A) Diagram of target sites of PBP1 and PBP3‐sgRNA. Target site of PBP1 is shown in the upper figure and target site of PBP3 is displayed in the bottom. Both target sites are at exon 2 (indicated with red region), containing a protospacer adjacent motif (PAM) sequence (TGG, in red). The restriction enzyme recognition sites are shown with the gray region and cutting sites are showed with an arrow. (B) Structure of elements of PBP1 and PBP3‐sgRNA. T7 promoter is underlined, the target sites with PAM sequence (TGG) removed are in box, and the guide RNA (gRNA) sequence is underlined by the dotted line.

According to the reported criteria of 5′‐GG‐(N)18‐NGG‐3′ (Hwang *et al*., 2013), the target sites of both CsupPBP1 and PBP3 (GenBank accession: ADK66921 and ADL09140) are located on exon 2 (Fig. 1A). The target site together with T7 promoter and guideRNA (gRNA) comprised the sgRNA sequence (Fig. 1B). The 3′‐NGG (protospacer adjacent motif [PAM] sequence) was removed from the target site sequence, and the 5′‐GG dinucleotide overlapped with the T7 promoter (Hwang *et al*., 2013). To produce the template DNA for *in vitro* transcription of sgRNA, forward primers PBP1‐sgR‐F‐1 and PBP3‐sgR‐F‐1 (Table S1) paired with the same reverse primer sgR‐R‐1 (Table S1) were used to clone these two genes, respectively (Bassett & Liu, 2014). Both forward primers contained T7 promoter, target site and part of the gRNA, while the 3′‐end of reverse primer overlapped part of the forward primer. The PCR conditions were same as that reported in Zhu *et al*. (2016).

The method for *in vitro* synthesis of Cas9 mRNA, PBP1‐ and PBP3‐sgRNA, including necessary kits and PCR conditions, refer to an earlier report from our laboratory (Zhu *et al*., 2016). The primers (sgR‐F‐2 and sgR‐R‐2) used for the amplification of *in vitro* transcription templates of two sgRNAs are shown in the Table S1.

### Cas9‐mRNA/sgRNA microinjection and mutation analysis

The pre‐experiment showed that microinjection with phosphate buffer had no toxic effect on larval growth and development. Cas9‐mRNA and PBP‐sgRNA were mixed at final concentrations of 300 ng/μL and 150 ng/μL, respectively, and the mixture (about 1 nL/egg) was injected into newly laid (less than 4 h old) eggs. A total of 397 eggs for PBP1 and 411 eggs for PBP3 were injected within 4 h, respectively. Injected eggs were incubated at 25°C 120 for 5–6 days until hatching.

After injection, around 40 injected eggs were collected 24 h later to detect possible mutation. The genomic DNA extracted (QIAamp DNA Mini Kit, Qiagen, Hilden, Germany) from these eggs was used as a template for polymerase chain reaction (PCR) amplification using PBP1‐F/R‐3 and PBP3‐F/R‐3 primers of both genes (Table S1). The PCR conditions were the same as that reported by Zhu *et al*. (2016), but the annealing temperature was 58°C and the extension time was 80 s in the present study. The PCR products were purified using AxyPrep PCP Cleanup Kit (Axygen, Suzhou, Jiangsu, China). The restriction enzymes *Aat*II for PBP1 and *Bsa*BI for PBP3 (New England Biolabs, Ipswich, MA, USA) that recognize the site adjacent to TGG (PAM sequence) were used for the restricted enzyme digestion and gel analysis (RED assay) to determine the potential mutations.

**Figure 2:**
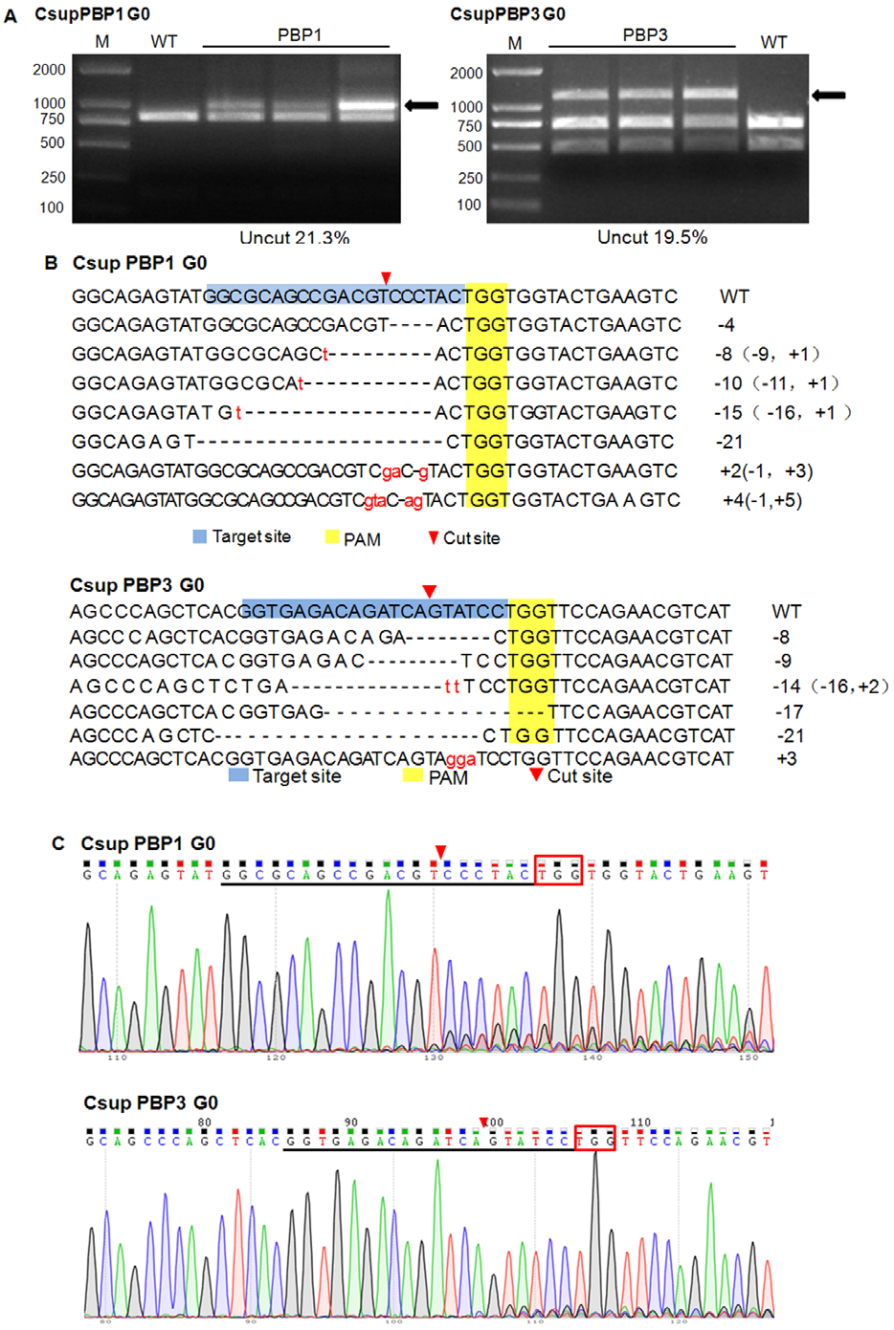
Detection of the CsupPBP1 (pheromone‐binding protein) and PBP3 mutation of injected eggs after 24 h. (A) The mutation efficiency of *PBP1* or *PBP3*, determined by the restricted enzyme digestion (RED) assay. M, Trans 2K DNA marker; WT, control group, using un‐injected eggs as templates; *PBP1* and *PBP3*, RED‐treated PCR products of the *PBP1* or *PBP3* gene from the genomic DNA of injected eggs. The black arrow indicates the uncleaved band. The percentage of the uncleaved band (21.3% and 19.5%) was calculated by Quantity One. (B) Mutant sequences at target site of *CsupPBP1* or *PBP3* in G0 eggs. The WT sequences are shown at the top with the target sites in blue region and protospacer adjacent motif (PAM) (TGG) in yellow region. In mutant sequences, deletions are shown as dashes and insertions as red lower case letters. (C) Representative sequencing chromatogram of polymerase chain reaction products from genomic DNA of injected G0 eggs. The top sequences indicate the WT sequences, with the target sites underlined and PAM (TGG) in box (arrows indicate the cutting sites of restriction enzymes), and the bottom chromatograms show multiple peaks, indicating the occurrence of the mutations.

PCR amplified fragments of two genes were first digested with designated enzymes and then were separated by gel electrophoresis in the RED assay (Beumer & Carroll, 2014). An uncleaved band would be shown if mutation occurred, and then mutation frequency was calculated by dividing uncleaved band intensity to the total band intensity from a single digestion experiment (Fig. 2A) (Guschin *et al*., 2010; Henriques *et al*., 2012). Band intensity was measured using Quantity One Software (Bio‐Rad, Hercules, CA, USA).

To determine the indel types of both genes, portions of PCR products containing the target site were cloned into the pEASY‐T3 cloning vector (TransGen, Beijing, China) following the manufacturer's instructions; then plasmid DNA was transformed into Trans‐T1 competent cells (TransGen, Beijing, China) and 15 positive clones of both genes were sequenced (Fig. 2B). Furthermore, portions of the PCR products were directly sequenced, and the multiple peaks at the target sites indicated the occurrence of mutations (Fig. 2C).

### Germline mutation transmission assay and mutant screening

To determine the transmission of the mutation induced by the CRISPR/Cas9 system, G0 adults were randomly single pair matched, allowing the mating between the male and female. Moth gDNAs that laid fertilized eggs were extracted individually using DNAiso Reagent (TaKaRa, Dalian, Liaoning, China), and then analyzed by RED assay. To obtain homozygous mutant germline, offspring from RED assay, positive parents were used for screening by an in‐cross strategy.

### Off target effect analysis

The potential off target sequences were searched by CasOT (Xiao *et al*., 2014) from a *C. suppressalis* genomic database (http://www.insect-genome.com/data/download.php) (Yin *et al*., 2014) in order to estimate the potential off target effects caused by Cas9/sgRNA injection. The top 10 potential off target sequences of each PBP gene were analyzed in six randomly selected G0 chimeras (Table S2). The amplification of DNA fragments that contained potential off target sites used the specific primer pairs (Table S1). The PCR products was purified by AxyPrep PCR Cleanup Kit (Axygen, Suzhou, Jiangsu, China), and directly sequenced.

### Electroantennogram (EAG) recordings

EAG values of PBP1 mutant males (five heterozygotes and seven homozygotes), PBP3 mutant males (12 heterozygotes and seven homozygotes) and eight wild type males to three sex pheromone components (*Z*11‐16:Ald, *Z*9‐16:Ald and *Z*13‐18:Ald) were recorded using the EAG instrument and related software (Syntech®, Hilversum, Netherlands). Four concentrations (0.1, 1, 10 and 100 ng/μL) for each pheromone component solution (with hexane as solvent), and 2‐day‐old virginal moths were used for the EAG recordings. The procedure for EAG recordings was the same as previously reported (Zhu *et al*., 2016).

### Statistical analysis

The difference in EAG values among WT, CsupPBP1^+/−^, CsupPBP1^−/−^, CsupPBP3^+/−^ and CsupPBP3^−/−^ males were analyzed by Duncan's new multiple range test, using SPSS 16.0 (SPSS Inc., Chicago, IL, USA).

## Results

### PBP mutations in eggs injected with sgRNA/Cas9

The gDNA extracted from 40 injected eggs was used to amplify the PBP fragment containing the target site. RED assay of the PCR product showed two additional bands on the gel, which indicated the occurrence of mutagenesis at the target site of the PBP sequence (Fig. 2A). Based on the relative intensities of the three bands, the indel (insert and deletion) frequency of *CsupPBP1* or *PBP3* gene was calculated as 21.3% and 19.5%, respectively.

To determine the indel sequences, portions of PCR products were subjected to the TA cloning and DNA sequencing (Fig. 2B). Seven of 15 sequenced clones from PBP1‐sgRNA‐injected eggs displayed diverse mutations, five clones with indel and two with deletion mutation, while six of 15 clones from PBP3‐sgRNA‐injected eggs displayed mutations, including four clones with deletion, one clone with indel, and one clone with insert mutation.

A portion of the PCR products from injected eggs was also directly sequenced (Fig. 2C). The multiple peaks at the target sites indicated the presence of mutations caused by injection of sgRNA/Cas9.

### Mutation heritability detection and homozygous mutant screening

After mutagenesis was detected, the remaining injected G0 eggs were reared on the laboratory conditions until adults (Table 1). Finally, 63 adults (28 males, 35 females) were obtained from PBP1‐sgRNA injected eggs, and 80 adults (37 males, 43 females) were obtained from PBP3‐sgRNA injected eggs.

**Table 1:** Statistical data of G0 insects after injection of pheromone‐binding protein (PBP)1 or PBP3‐single guide RNA (sgRNA)/Cas9 messenger RNA

Gene	gRNA/Cas9 concentration (ng/μL)	No. of injected embryos	Hatching rate (%)	No. of larvae†	No. of pupae	Pupation rate (%)
*CsupPBP1*	150/300	397	53.9	111	63 (35♀/28♂)	56.7
*CsupPBP3*	150/300	411	53.7	133	80 (43♀/37♂)	60.1
*CsupPBS*		364	41.7	101	68 (31♀/37♂)	67.3

^†^No. of larvae was counted at 15th day after the egg hatching.

**Figure 3:**
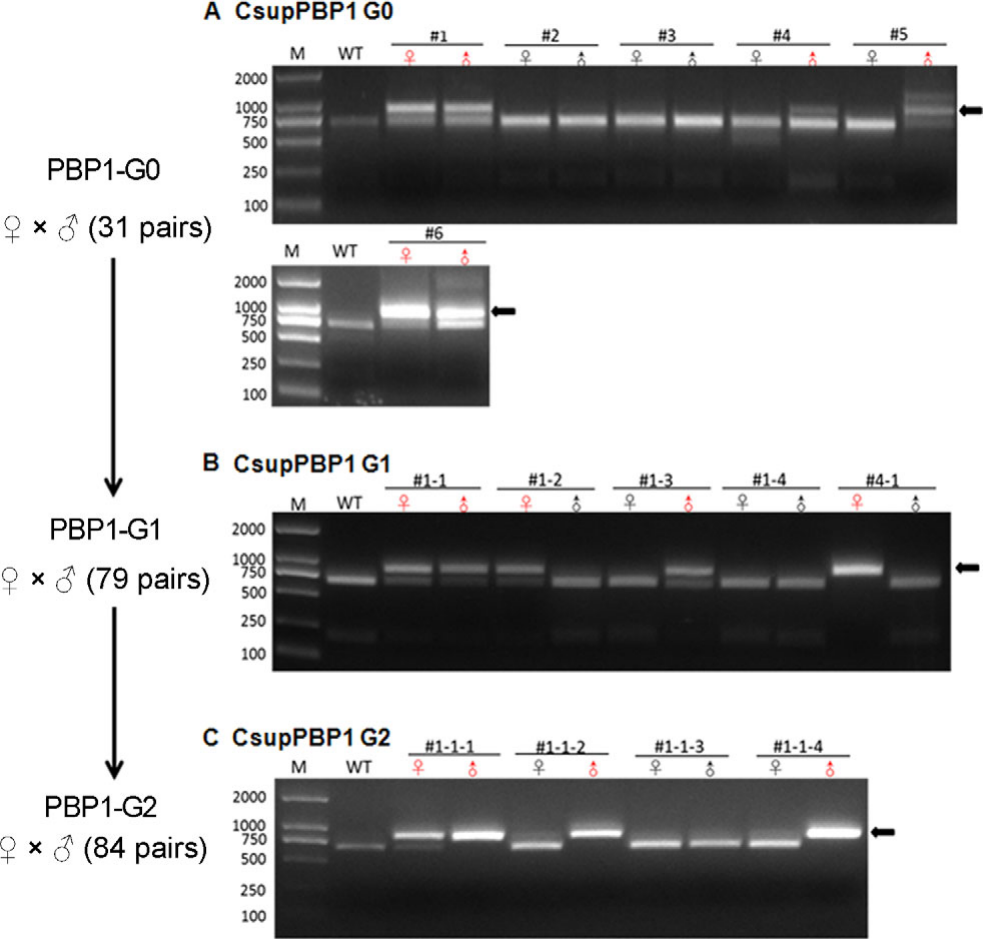
Genotype detection of CsupPBP1 (pheromone‐binding protein) germlines that laid fertilized eggs, by restricted enzyme digestion (RED) assay. (A), (B) and (C) indicate the detection in G0, G1 and G2 insects, respectively. The single pair numbers are shown on the top of the gel picture; mutant males (♂) and females (♀) are in red. M, DNA Maker; WT, wild‐type moths; the black arrows point to the uncleaved band, indicating mutants occurred.

To screen the homozygous mutants, G0 adults were in‐crossed with each other. In total, 31 single pairs were made using adults from PBP1‐sgRNA injected eggs and three wild type males, of which six single pairs successfully mated and generated G1 offspring. RED assay showed that one or both G0 parents of four single pairs were positive (Fig. 3A), thus G1 offspring from these four single pairs were carefully raised and in‐crossed to establish mutant lines. Again, five single pairs successfully generated PBP1‐G2 offspring and four single pairs of G1 animals were RED assay positive (Fig. 3B).

To determine the genotype of G1 moths that laid fertilized eggs, a portion of PCR products was subjected to the TA clone sequencing. Two different deletions of 16 and 17 bp in PBP1‐G1 animals were obtained (Fig. S1). Compared to the wild type of 167 amino acids in length (Fig. S2 A), the two mutant genotypes had only 119 and 82 amino acids, respectively, due to introduction of a terminator codon (TGA) (Fig. S2 B, C). The mutant PBP1 with 82 amino acids contained only one conserved cysteine, suggesting a complete loss of the normal function, and therefore, this mutant genotype (#1‐1) was used for further screening. The PBP1‐G2 mutants were in‐crossed by single pairs to generate PBP1‐G3 offspring. Among PBP1‐G2 animals, three pairs that laid eggs were checked to be positive (Fig. 3C), and heterozygous or homozygous PBP1‐G2 animals were obtained. The offspring of #1‐1‐1 germline were used for electrophysiological assay.

**Figure 4:**
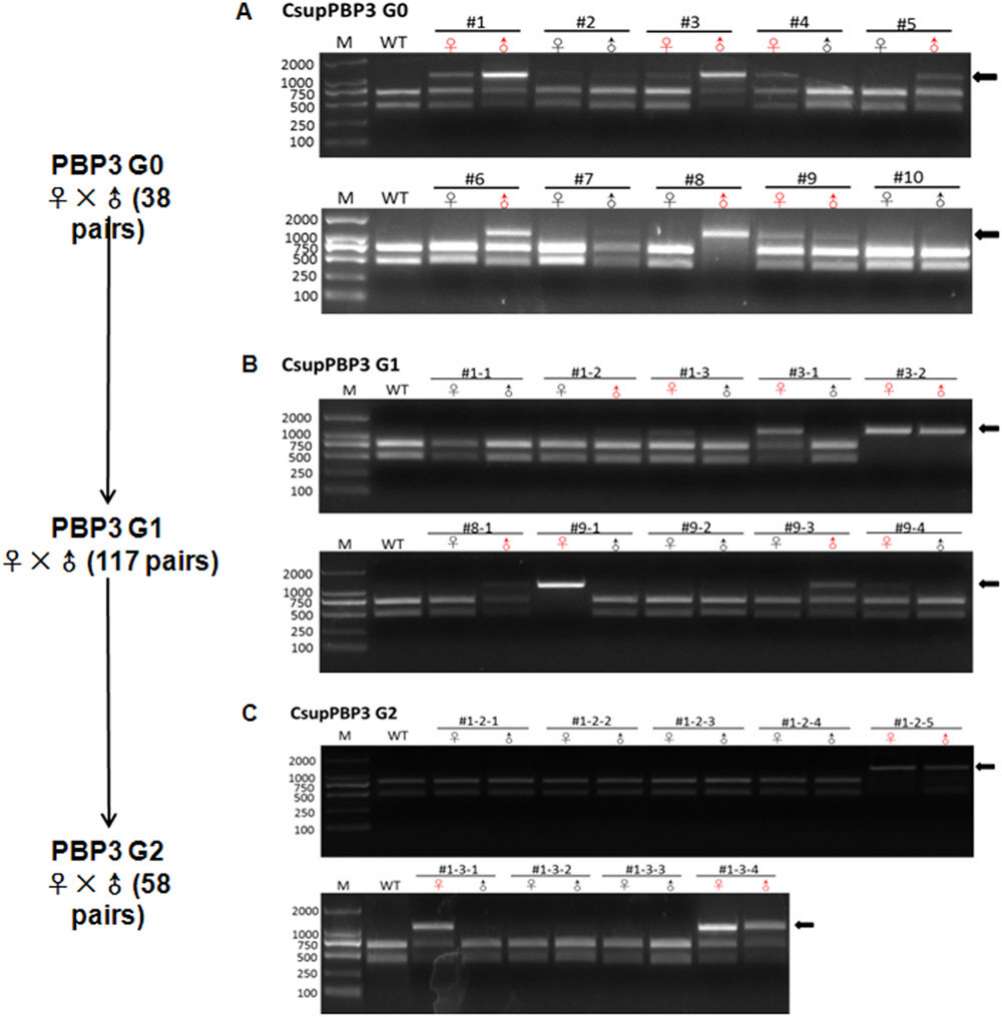
Genotype detection of CsupPBP3 (pheromone‐bind protein) germlines that laid fertilized eggs, by restricted enzyme digestion (RED) assay. (A), (B) and (C) indicate the detection in G0, G1 and G2 insects, respectively. The single pair numbers are shown on the top of the gel picture; mutant males (♂) and females (♀) are in red. M, DNA Maker; WT, wild type moths; the black arrows point to the uncleaved band, indicating mutants occurred.

A similar procedure was used to screen the homozygous PBP3 mutants. A total of 38 single pairs of G0 adults were set, and 10 pairs generated G1 offspring. Among these 10 pairs, eight pairs (with one or two parents) were tested to be positive by RED assay (Fig. 4A). Further, 10 pairs of PBP3‐G1 adults generated PBP3‐G2 offspring, in which eight pairs were RED assay positive (Fig. 4B). At this stage, G1 adults of the eight pairs were checked for PBP3 genotype by the TA clone sequencing. Two different deletions of 7 and 12 bp in PBP1‐G1 animals were detected (Fig. S3). Compared to 166 amino acids in length for the wild type (Fig. S4A), the mutant PBP3s with 7 bp and 12 bp deletion had only 69 and 162 amino acids, respectively, due to introduction of a terminator codon (TGA) (Fig. S4 B, C). The mutant PBP3 with 69 amino acids (#1‐2 and #1‐3) contained only one conserved cysteine, suggesting a complete loss of the normal function, and therefore, was used for further screening. The PBP3‐G2 mutants were in‐crossed by single pairs to generate PBP1‐G3 offspring (Fig. 4C). The offspring of #1‐3‐4 germline were used for electrophysiological assay.

### EAG responses of PBP mutant males

**Figure 5:**
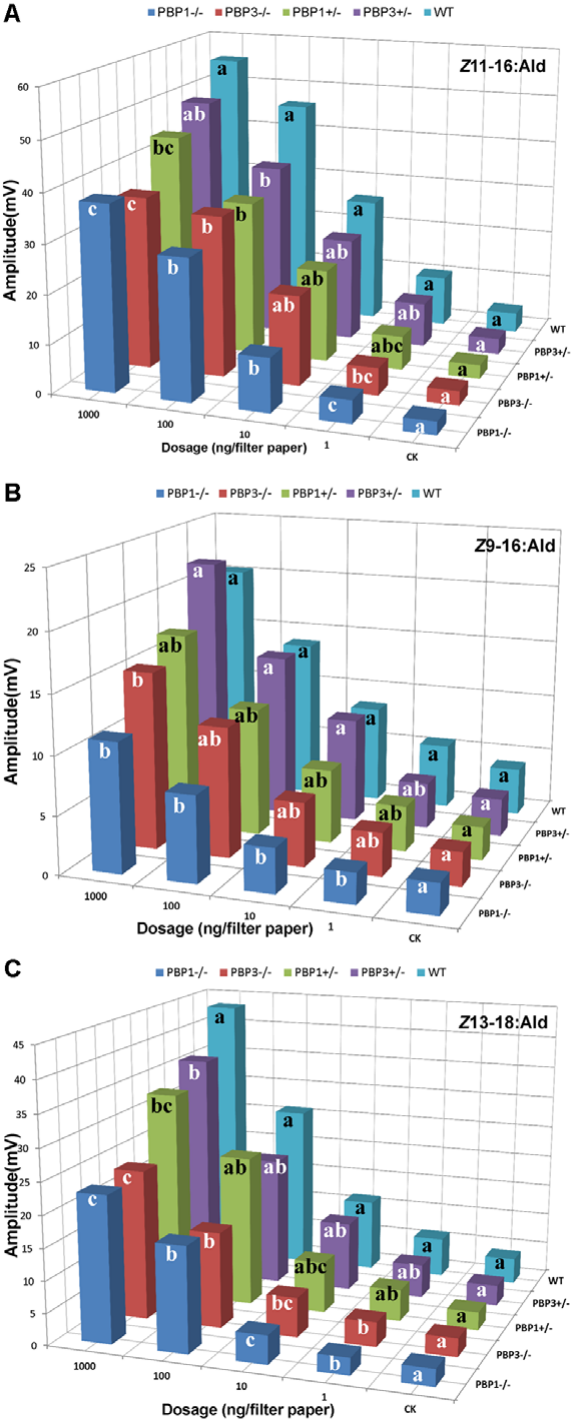
Electroantennogram (EAG) response of CsupPBP1 (pheromone‐binding protein) and PBP3 homozygous and heterozygous mutant males to three sex pheromone components at different dosages. WT, wild type moths (*n* = 8); PBP1+/−, PBP1 heterozygotes (*n* = 5); PBP1−/−, PBP1 homozygotes (*n* = 7); PBP3+/−, PBP3 heterozygotes (*n* = 12); PBP3−/−, PBP3 homozygotes (*n* = 7). CK, the control (hexane). Different letters on columns of same dosage indicate significant difference in EAG values (Duncan's new multiple range test, *P* < 0.05).

The EAG responses of wild type, heterozygous and homozygous mutant males to sex pheromones at different dosages were measured (Fig. 5 and Table S3). Compared with wild type males, homozygous *PBP1*‐mutant males showed significantly lower EAG responses to all three pheromone components even at the dosage of 1 ng. A similar phenomenon was observed in heterozygous mutant males, but the extent of the reduction in EAG response was less than the homozygous mutants. Heterozygous mutant males showed significantly decreased EAG responses only at high pheromone dosages, 100 ng for *Z*11‐16:Ald, 1000 ng for *Z*13‐18:Ald (Fig. S5), and showed no significant reduction in EAG responses to *Z*9‐16:Ald at any tested dosage.

As for *PBP3* mutants, homozygous mutant males display significant decrease in EAG response at all tested dosages for the major component *Z*11‐16:Ald, but only at higher dosages (10–1000 ng) for the two minor components, while heterozygous mutants did not show significant reduction at the lower dosages of 1–10 ng for *Z*11‐16:Ald and *Z*13‐18:Ald, and at any dosages for *Z*9‐16:Ald.

## Discussion

With four different PBP genes found in *C. suppressalis* (Cao *et al*., 2014), functional differentiation among them comes as an important point for better understanding the complexity and sensitivity of the sex pheromone olfactory system. In this study, we for the first time provide direct *in vivo* evidence that both CsupPBP1 and 3 function in the sex pheromone perception, and that CsupPBP1 is more important than CsupPBP3, based on the electrophysiological response to the sex pheromones.

Previously, expression profile (such as antennal specificity, expression level in male antennae, male/ female ratio of antennal expression), and *in vitro* binding affinity for pheromone components were determined in some moths including *C. suppressalis*. The results in *C. suppressalis* showed that among the four PBPs, *PBP1* is the most male‐biased and male‐abundant in RNA expression (Chang *et al*., 2015), suggesting PBP1 plays the most important role in sex pheromone perception, given that relative expression levels reflect the relative importance or environmental abundance of their ligands (Baker *et al*., 2012). Ligand binding assays showed that PBP1 and PBP3 had much higher binding affinity than two other PBPs (PBP2 and PBP4), and did not show obvious selectivity among three pheromone components (Chang *et al*., 2015). Our results of the present *in vivo* functional study are generally in agreement with the above literature reported. First, males with PBP1 or PBP3 knocked out showed significant decrease in EAG response to all three pheromone components, demonstrating that both PBPs functions in perception of sex pheromones, but without obvious component selectivity. Similar non‐selectivity was also found in other moths such as *Spodoptera exigua* (Liu *et al*., 2015), *S. litura* (Liu *et al*., 2012a) and *Helicoverpa armigera* (Guo *et al*., 2012), by ligand binding assay. Therefore, moth PBPs at least in some species, are unable to recognize different components of the sex pheromone blend. It has been well demonstrated that ORs play the key role in recognition of different odorants and pheromone components (Sakurai *et al*., 2004; Grosse‐Wilde *et al*., 2006; Chang *et al*., 2015).

With regard to the relative importance, our present study indicates that PBP1 is more important than PBP3 in *C. suppressalis*. In the EAG assay, PBP1 mutant males displayed lower EAG response than PBP3 mutant males, especially at lower test dosages. Taking 10 ng usage for example, the EAG values of PBP1 mutants were 40.0%, 30.4% and 27.7% lower than that of PBP3 mutants to components *Z*11‐16:Ald, *Z*9‐16:Ald and *Z*13‐18:Ald, respectively. However, this reduction in EAG response is not comparable with the difference in their relative expression, as *PBP1* level in male antennae is as high as three‐fold that of *PBP3* (Chang *et al*., 2015). This might be due to compensation by other PBPs and even OBPs and CSPs. *In vitro* ligand binding assays suggested that general odorant binding proteins (GOBPs, particularly GOBP2) may function to transport sex pheromones in *C. suppressalis* (Gong *et al*., 2009; Khuhro *et al*., 2017) and some other moths (Jacquinjoly *et al*., 2000; Zhou *et al*., 2009; He *et al*., 2010; Liu *et al*., 2012b); an antenna‐predominant and male‐biased CSP19 in *Sesamian inferens* showed high binding affinities to female sex pheromones with Ki values comparable to that of PBPs, suggesting its involvement in sex pheromone perception (Zhang *et al*., 2014). Furthermore, interactions among OBPs in ligand binding were found in *Holotrichia oblita* (Wang *et al*., 2013a). The compensation and interactions among PBPs and even other binding proteins need to be further clarified. It is noted that *Drosophila melanogaster*, with loss‐of‐function of a single PBP (lush), was completely devoid of evoked activity to its pheromone component (Xu *et al*., 2005), indicating variability of the pheromone perception pathway between dipteran and lepidopteran species.

The heterozygous *PBP1* or *PBP3* mutant males were also tested in the present study, suggesting the mutation is an incomplete dominant mutation. Heterozygous *PBP1* or *PBP3* mutants showed EAG values lower than wild type males, but higher than homozygous mutant males. This incomplete dominance of mutation is similar as that with *PBP1* knockout in *H. armigera* (Ye *et al*., 2017). In contrast, CRISPR/Cas9 inducted gene knockout led to recessive mutations with *Orco* gene (Olfactory receptor co‐receptor) in *S. littoralis* (Koutroumpa *et al*., 2016) and *BLOS2* gene in the silkworm (Ma *et al*., 2012), suggesting different regulation of gene expression.

We tried to maintain both *PBP1* and *PBP3* homozygous mutant lines, but unfortunately, G3 homozygous adults displayed a very short life of only 2 days, which led to a failure in mating and laying fertilized eggs. Failure in maintenance of the homozygous line has also been reported for knockout of *Orco* in *Ostrinia furnacalis* by TALEN (Yang *et al*., 2015) and *PBP3* in *S. litura* by CRISPR/Cas9 (Zhu *et al*., 2016). The reasons might be multiple and various between studies. In the two studies above, it was likely attributed to reduction in mating successes for those adults with *Orco* or *PBP1* knocked out. However, in the present study we cannot exclude the off‐target effects that the CRISPR/Cas9 system induces. First, the vitality is unlikely affected by or directly related to PBP genes. Second, off‐target effects have been reported in studies by using this system (Cradick *et al*., 2013; Pattanayak *et al*., 2013). We did not observe any mutation in the top 10 potential sequences, but the potential off target sequences were identified using a *C. suppressalis* genomic data of low quality (http://www.insect-genome.com/data/download.php), which is the only genomic data available. It is possible that sequences of higher off target potentiality might exist. With complete and more accurate genome data, we may determine whether this shortened moth longevity results from the off target effect.

In conclusion, high rate of mutagenesis targeting *CsusPBP* was induced by direct egg injection of PBP‐sgRNA/Cas9‐mRNA, and homozygous mutant insects were obtained in G3 generation using an in‐cross strategy. EAG assay with PBP mutant moths demonstrated that both PBP1 and PBP3 play an important role in the sex pheromone perception of *C. suppressalis*, but PBP1 is more important than PBP3. Our study provides the important direct evidence for the roles of PBP1 and PBP3 in sex pheromone perception of *C. suppressalis*, and a valuable methodological reference for gene functional study in other genes and in other moth species.

## Disclosure

The authors have no conflict of interests to declare.

## Supporting information


**Table S1** The primers used in the study.
**Table S2** The top 10 potential off target (OT) sequences.
**Table S3** PBP1 or PBP3 knocked out induced significantly decrease of EAG response to three sex pheromones at different dosages.

**Fig. S1** Representative chromatograms of PBP1 PCR products amplified by the gDNA from G1 moths that laid fertilized eggs. (A), (B) and (C) show PBP1 heterozygotes with 17 bp deletion, PBP1 homozygote with 17 bp deletion, and PBP1 heterozygotes with 16 bp deletion, respectively. The stacked peaks indicate the heterozygotes, by direct sequencing of the PCR products; WT and “−17” or “−16” show the wild type and the mutant (a 17 bp or 16 bp deletion) sequences respectively, which are determined by TA cloning and sequencing. The target site is underlined.

**Fig. S2** Amino acid sequence of PBP1 wild type (A) and G1 mutant (B and C) moths. The conserved cysteines (C) are boxed, showing six cysteines in the wild type sequence, and only one or two cysteines in the mutant sequences.

**Fig. S3** Representative chromatograms of PBP3 PCR products amplified by the gDNA from G1 moths that laid fertilized eggs. (A), (B) and (C) show PBP3 heterozygotes with 7 bp deletion, PBP3 homozygote with 12 bp deletion and PBP3 heterozygotes with 12 bp deletion, respectively. The stacked peaks indicate the heterozygotes, by direct sequencing of the PCR products; WT and “−7” or “−12” show the wild type and the mutant (a 7 bp or 12 bp deletion) sequences respectively, which are determined by TA cloning and sequencing. The target site is underlined.

**Fig. S4** Amino acid sequence of PBP3 wild type (A) and G1 mutant (B and C) moths. The conserved cysteines (C) are boxed, showing six cysteines in the wild type sequence and the mutant sequence with 12 bp deletion, and only one cysteine in the mutant sequences with 7 bp deletion.

**Fig. S5** Typical EAG response diagrams of PBP1 wild type (A), PBP1+/− (B) and PBP1−/− (C) males to *Z*13–18:Ald (1000 ng). The EAG value for wild type, PBP1+/− and PBP1−/− males are 47.408, 30.436 and 26.589 mV, respectively. The scale of the schematics is 5 mV.
