# Supplementary material for: CRISPR/Cas9‐mediated PBP1 and PBP3 mutagenesis induced significant reduction in electrophysiological response to sex pheromones in male Chilo suppressalis
Source: Insect Sci. 2017 Dec 7;26(3):388–99. doi: 10.1111/1744-7917.12544 (PMC7379591; doi:10.1111/1744-7917.12544)
Supplement: Supplementary file 1 — Table S1 The primers used in the study. Table S2 The top 10 potential off target (OT) sequences. Table S3 PBP1 or PBP3 knocked out induced significantly decrease of EAG response to three sex pheromones at different dosages. [file INS-26-388-s002.pdf]

Table S1 The primers used in the study

Primer name	Sequence (5'-3')	Purpose
PBP1-sgR-F-1	TAATACGACTCACTATAGGCGCAGCCGACGTCCCTACGTTTTAGAGCTAGAAATAGCAAGTTAAAATAA	Synthetic sgRNA template
PBP3-sgR-F-1	TAATACGACTCACTATAGGTGAGACAGATCAGTATCCGTTTTAGAGCTAGAAATAGCAAGTTAAAATAA	Synthetic sgRNA template
sgR-R-1	AAAAGCACCGACTCGGTGCCACTTTTTCAAGTTGATAACGGACTAGCCTTATTTTAACTTGCTATTTCTAGCTCTAA	Synthetic sgRNA template
sgR-F-2	CGGTGATGACGGTGAAAACCTC	Synthetic sgRNA template
sgR-R-2	AGCACCGACTCGGTGCC	Synthetic sgRNA template
PBP1-F-3	CGCCATTTCCTCACCTGATAGT	Positive gene knockout insect screening
PBP1-R-3	GCAACATTTAATTTGTCCCATG	Positive gene knockout insect screening
PBP3-F-4	AAGGGCTTTTGCTCTCTCATACT	Positive gene knockout insect screening
PBP3-R-4	GCCTACAACAACGCATTTCG	Positive gene knockout insect screening
PBP1-OT1-F	TAGCACACTCCCTGTCCCT	Check the potential off target effects
PBP1-OT1-R	GTGGACACACGAGTATTTGG	Check the potential off target effects
PBP1-OT2-F	ACCCTACAACCAACACACG	Check the potential off target effects
PBP1-OT2-R	ATAAAGTTTCACTGCTCAATCC	Check the potential off target effects
PBP1-OT3-F	TTGTTTGGTTTCGCATTCAC	Check the potential off target effects
PBP1-OT3-R	TGGGACGGAAACTAAACTGC	Check the potential off target effects
PBP1-OT4-F	GGAACTAACGCCGCCATCT	Check the potential off target effects
PBP1-OT4-R	TCGAACTGAAAACCCGCTATC	Check the potential off target effects
PBP1-OT5-F	GTACTGCCTTTTCGTCCCT	Check the potential off target effects
PBP1-OT5-R	AGACTCCCACTCGCTAACG	Check the potential off target effects
PBP1-OT6-F	AACTCAGGCATAGCAACGG	Check the potential off target effects
PBP1-OT6-R	CGTGACCATAATGATCTTATCG	Check the potential off target effects
PBP1-OT7-F	TAGTTAGCCGTTGTCGTGG	Check the potential off target effects
PBP1-OT7-R	CTCAAATCGGTCCCAACTC	Check the potential off target effects
PBP1-OT8-F	TAAGAACTGATTCAAAAGCGA	Check the potential off target effects

PBP1-OT8-R	CATAGAAGATAACTTGCGGCT	Check the potential off target effects
PBP1-OT9-F	TCCCATAAAACATACACGCAT	Check the potential off target effects
PBP1-OT9-R	CAGAACGAAAAGCAGCCTC	Check the potential off target effects
PBP1-OT10-F	GCAGAGCCATCTATGTTGAGC	Check the potential off target effects
PBP1-OT10-R	GGGCGAAAGGGATTTACTC	Check the potential off target effects
PBP3-OT1-F	AGTGCTTCCCAATGACCGT	Check the potential off target effects
PBP3-OT1-R	GATGTTGGGGTGGGGACTA	Check the potential off target effects
PBP3-OT2-F	CGAATGAAGTCACCGCTAA	Check the potential off target effects
PBP3-OT2-R	TTGGTCCGTGCTAAGGGTA	Check the potential off target effects
PBP3-OT3-F	TCTGTGTCCCTGTCCATCA	Check the potential off target effects
PBP3-OT3-R	TGGCGAATCAAAGTCAACA	Check the potential off target effects
PBP3-OT4-F	TAATCCCGAAAAAAGGTAG	Check the potential off target effects
PBP3-OT4-R	GATGAGGTACTCGCACAAG	Check the potential off target effects
PBP3-OT5-F	TATTCTGTAGGTGGAGGCA	Check the potential off target effects
PBP3-OT5-R	TAACTACTGAAAGACGGGC	Check the potential off target effects
PBP3-OT6-F	CCTGCGAGGTTTCTGGTAT	Check the potential off target effects
PBP3-OT6-R	GGCGGTTGAAGTAAAAGGA	Check the potential off target effects
PBP3-OT7-F	GCCTCTTTGTGACTCTGGA	Check the potential off target effects
PBP3-OT7-R	ACTGGATGGTGAAGCAAAA	Check the potential off target effects
PBP3-OT8-F	TCTTCGTCCCAACCAATGC	Check the potential off target effects
PBP3-OT8-R	CTGAGATGCTCCAAAGACCCA	Check the potential off target effects
PBP3-OT9-F	AAATGTTACCTGCGACCAA	Check the potential off target effects
PBP3-OT9-R	CTACCTTTGTCAGAGTGTTGTT	Check the potential off target effects
PBP3-OT10-F	CTACAAATCGGGCGACAAA	Check the potential off target effects
PBP3-OT10-R	AGAGGCTAAGGGTGCCAATAC	Check the potential off target effects

Table S2 The top 10 potential off target (OT) sequences

Sequence NO.	Location in the DNA database	Off target site	Mismatch type	Mismatch number
PBP1-OT1	csug3874:10956-10979	GGgtaAGg_CGACGTCCtTAC-CGGG	A14	5
PBP1-OT2	csug5366:8665-8688	GGtcCcGt_CGACGTCCCaAC-AGGG	A14	5
PBP1-OT3	csug88340:2730-2753	GGgtaAGg_CGACGTCCtTAC-CGGG	A14	5
PBP1-OT4	csug148230:2273-2296	aaCGataC_CGAtGTCCCTAC-TGGT	A15	6
PBP1-OT5	csug8246:2160-2183	cctGacaC_CGACGTCCCgAC-GGGT	A16	7
PBP1-OT6	csug80469:2492-2515	tctcCtcC_CGACGTCCtTAC-TGGT	A16	7
PBP1-OT7	csug100820:1672-1695	caaagcag_CGgCGTCCCTAC-CGGA	A18	9
PBP1-OT8	csug2948:19514-19537	GGgaaAGC_CtACtTCCCTAC-TGGC	A23	5
PBP1-OT9	csug12454:7477-7500	GGgGCgGg_CGACGTgCCaAC-TGGT	A23	5
PBP1-OT10	csug77148:8518-8541	cGCGatGC_CGACGTgtCTAC-GGGC	A23	5
PBP3-OT1	csug175899:6073-6096	GcacAGAt_AGcTCAGTATCC-CGGC	A14	5
PBP3-OT2	csug601:11117-11140	ttTtgaAC_AGATCAtTATCC-GGGT	A15	6
PBP3-OT3	csug238466:2874-2897	aGatAGca_AGATCAGTtTCC-AGGT	A15	6
PBP3-OT4	csug243255:3040-3063	GaaaAtAa_AGATCcGTATCC-GGGG	A15	6
PBP3-OT5	csug2070:23824-23847	taTctcAg_AaATCAGTATCC-CGGG	A16	7
PBP3-OT6	csug23393:4283-4306	atgaAagC_AGATCAtTATCC-TGGA	A16	7
PBP3-OT7	csug33288:1861-1884	GcgtgagC_AGATCAGTAaCC-AGGT	A16	7
PBP3-OT8	csug36886:7846-7869	tGgtcccC_AGATCtGTATCC-AGGT	A16	7
PBP3-OT9	csug57622:1880-1903	atTctatC_AGATCAaTATCC-AGGT	A16	7
PBP3-OT10	csug126041:1216-1239	cGaaAtgt_AGATCAGTATaC-TGGC	A16	7

The off target sites are presented as **<non-seed>_<seed>-<PAM>**, in which lower-case letters in the protospacer indicate mismatched nucleotides . For mismatch type, “A14” means the PAM level is A (NGG), the counts of mismatches in the seed and non-seed regions are 1 and 4, respectively. Mismatch number: count of mismatches in the protospacer.

Table S3 PBP1 or PBP3 knocked out induced significantly decrease of EAG response to three sex pheromone at different dosage

Germline		Z9-16:Ald				Z11-16:Ald				Z13-18:Ald			
	CK	1	10	100	1000	1	10	100	1000	1	10	100	1000
**WT**	4.07 ±0.40 a	5.56 ±1.02 a	8.43 ±3.41 a	13.86 ±2.76 a	20.28 ±2.45 a	10.32 ±1.50 a	25.87 ±4.12 a	46.08 ±3.50 a	55.36 ±3.21 a	6.23 ±1.17 a	11.41 ±3.50 a	25.98 ±3.54 a	43.27 ±3.19 a
**PBP1$^{-/-}$**	2.67 ±0.23 a	2.65 ±1.47 b	3.84 ±3.50 b	7.42 ±2.83 b	11.10 ±2.51 b	4.73 ±1.52 c	11.02 ±4.22 b	28.77 ±3.59 b	37.79 ±3.30 c	2.70 ±1.19 b	4.50 ±3.59 c	16.58 ±3.63 b	22.96 ±3.27 c
**PBP3$^{-/-}$**	2.92 ±0.31 a	3.74 ±1.47 ab	5.52 ±3.50 ab	11.17 ±2.83 ab	15.21 ±2.51 b	5.61 ±1.52 bc	18.37 ±4.22 ab	32.84 ±3.59 b	35.22 ±3.30 c	3.86 ±1.19 b	6.22 ±3.59 bc	15.05 ±3.63 b	23.54 ±3.27 c
**PBP1$^{+/-}$**	2.85 ±0.28 a	3.92 ±1.13 ab	6.35 ±3.86 ab	10.99 ±3.04 ab	16.95 ±2.70 ab	6.94 ±1.64 abc	19.06 ±4.54 ab	31.68 ±3.86 b	44.15 ±3.54 bc	4.98 ±1.28 ab	8.07 ±3.85 abc	23.70 ±3.90 ab	32.87 ±3.51 bc
**PBP3$^{+/-}$**	3.30 ±0.38 a	3.99 ±1.13 ab	9.00 ±3.25 a	14.07 ±2.63 a	22.04 ±2.33 a	9.14 ±1.41 ab	21.22 ±3.92 ab	35.55 ±3.33 b	48.64 ±3.06 ab	5.34 ±1.11 ab	10.98 ±3.33 ab	20.39 ±3.37 ab	36.17 ±2.86 b

EAG values (Mean ± SE) in the same column followed with the same letter were not significantly different (ANOVA followed by Duncan's new multiple range test, $P < 0.05$). WT, wild type moths (n=8); PBP1$^{-/-}$, homozygote moths of PBP1 (n=7); PBP3$^{-/-}$, homozygote moths of PBP3 (n=7); PBP1$^{+/-}$, heterozygote moths of PBP1 (n=5); PBP3$^{+/-}$, heterozygote moths of PBP3 (n=12).

Figure Legend

Fig. S1 Representative chromatograms of PBP1 PCR products amplified by the gDNA from G1 moths that laid fertilized eggs

(A), (B) and (C) show PBP1 heterozygotes with 17 bp deletion, PBP1 homozygote with 17 bp deletion, and PBP1 heterozygotes with 16 bp deletion, respectively. The stacked peaks indicate the heterozygotes, by direct sequencing of the PCR products; WT and "-17" or "-16" show the wild type and the mutant (a 17 bp or 16 bp deletion) sequences respectively, which are determined by TA cloning and sequencing. The target site is underlined.

Fig. S2 Amino acid sequence of PBP1 wild type (A) and G1 mutant (B and C) moths

The conserved cysteines (C) are boxed, showing 6 cysteines in the wild type sequence, and only one or two cysteines in the mutant sequences.

Fig. S3 Representative chromatograms of PBP3 PCR products amplified by the gDNA from G1 moths that laid fertilized eggs

(A), (B) and (C) show PBP3 heterozygotes with 7 bp deletion, PBP3 homozygote with 12 bp deletion and PBP3 heterozygotes with 12 bp deletion, respectively. The stacked peaks indicate the heterozygotes, by direct sequencing of the PCR products; WT and "-7" or "-12" show the wild type and the mutant (a 7 bp or 12 bp deletion) sequences respectively, which are determined by TA cloning and sequencing. The target site is underlined.

Fig. S4 Amino acid sequence of PBP3 wild type (A) and G1 mutant (B and C) moths

The conserved cysteines (C) are boxed, showing 6 cysteines in the wild type sequence and the mutant sequence with 12 bp deletion, and only one cysteine in the mutant sequences with 7 bp deletion.

Fig. S5 Typical EAG response diagrams of PBP1 wild type (A), PBP1+/- (B) and PBP1-/- (C) males to *Z*13-18:Ald (1000 ng)

The EAG value for wild type, PBP1+/- and PBP1-/- males are 47.408, 30.436 and 26.589mV, respectively. The scale of the schematics is 5mV.
