# Supplementary figures and images for: CRISPR/Cas9‐mediated PBP1 and PBP3 mutagenesis induced significant reduction in electrophysiological response to sex pheromones in male Chilo suppressalis
Source: Insect Sci. 2017 Dec 7;26(3):388–99. doi: 10.1111/1744-7917.12544 (PMC7379591; doi:10.1111/1744-7917.12544)

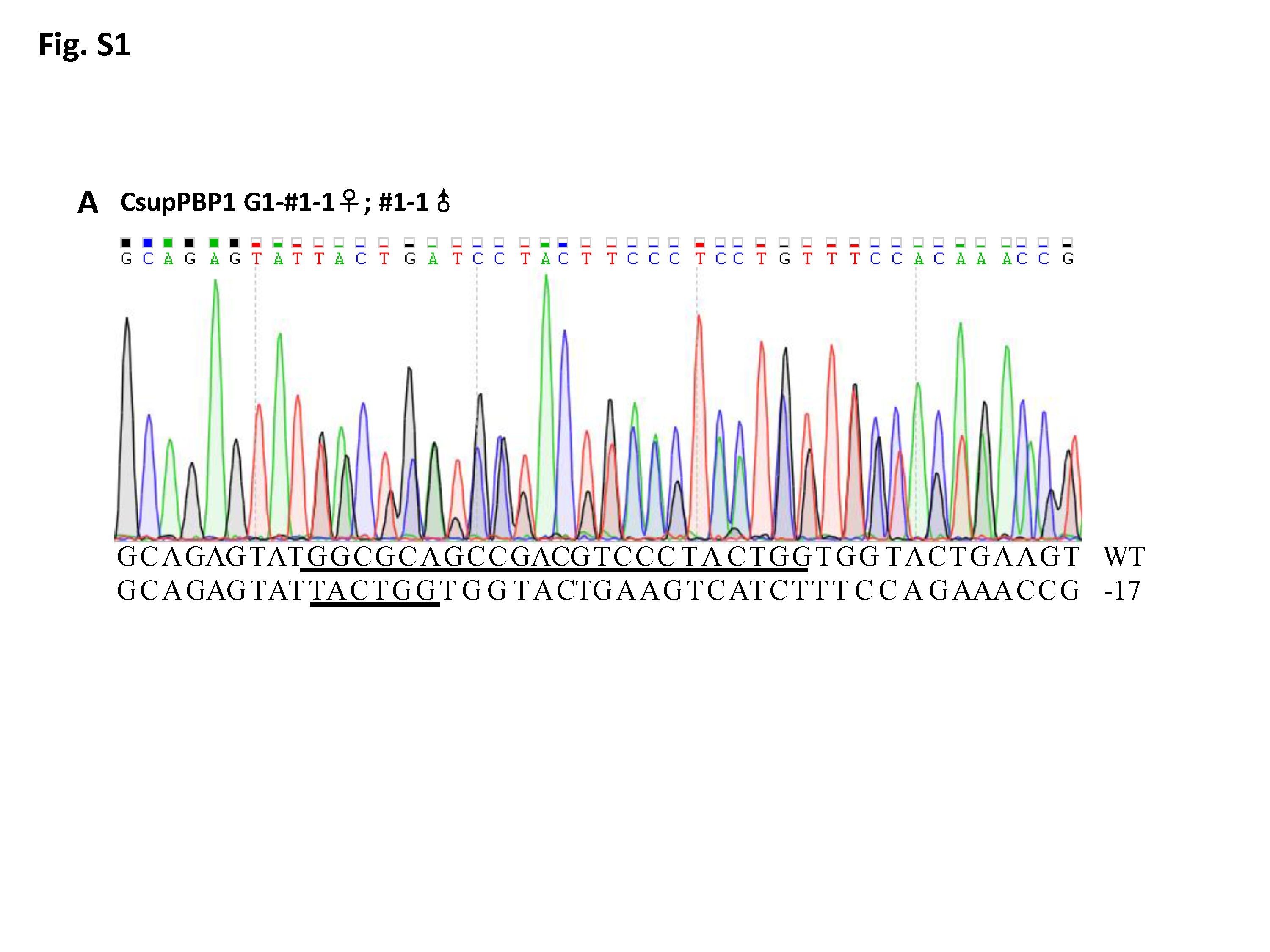

Supplement: Supplementary file 2 — Fig. S1 Representative chromatograms of PBP1 PCR products amplified by the gDNA from G1 moths that laid fertilized eggs. (A), (B) and (C) show PBP1 heterozygotes with 17 bp deletion, PBP1 homozygote with 17 bp deletion, and PBP1 heterozygotes with 16 bp deletion, respectively. The stacked peaks indicate the heterozygotes, by direct sequencing of the PCR products; WT and “−17” or “−16” show the wild type and the mutant (a 17 bp or 16 bp deletion) sequences respectively, which are determined by TA cloning and sequencing. The target site is underlined. [file INS-26-388-s008.tiff]

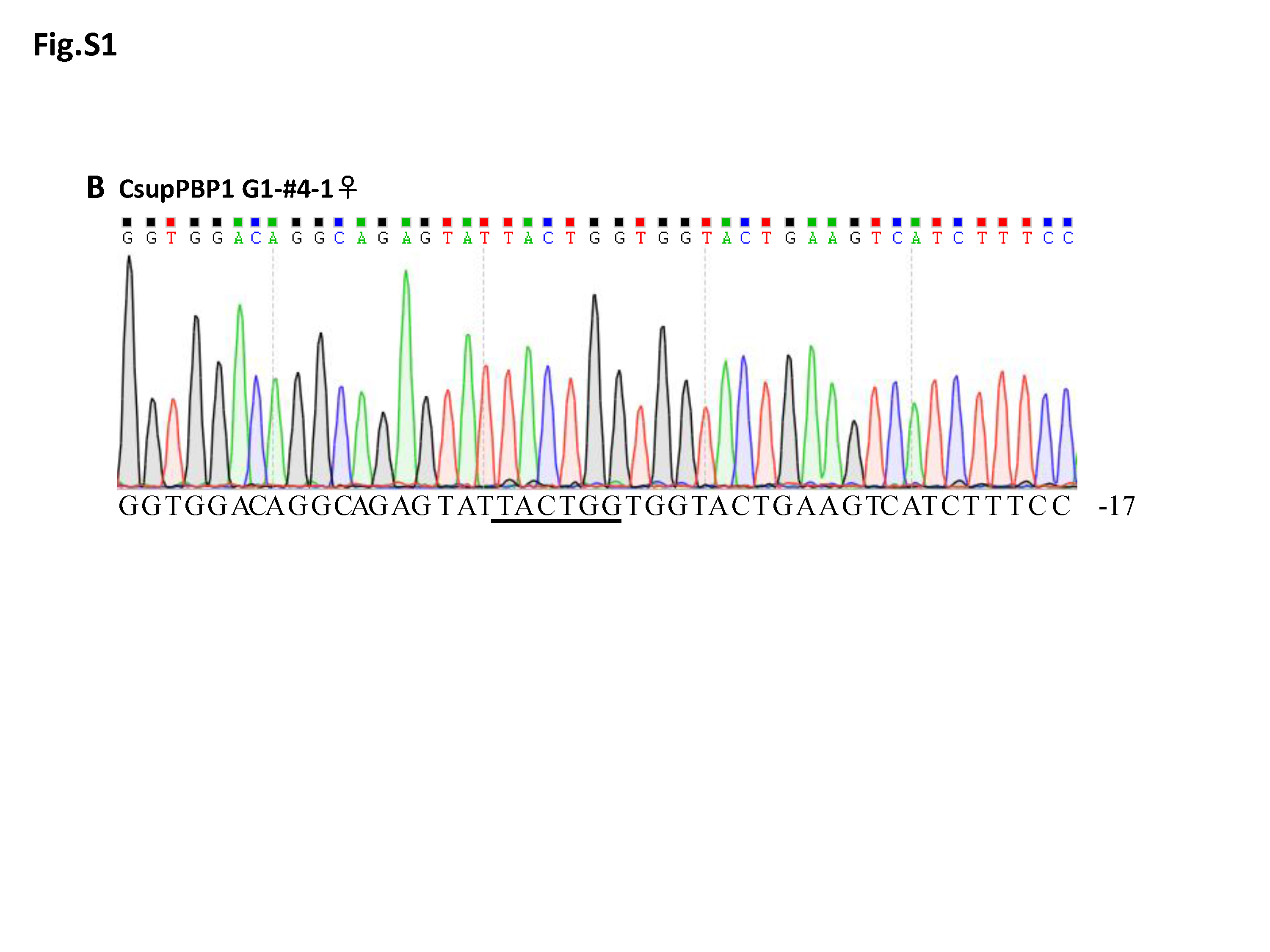

Supplement: Supplementary file 3 [file INS-26-388-s009.tiff]

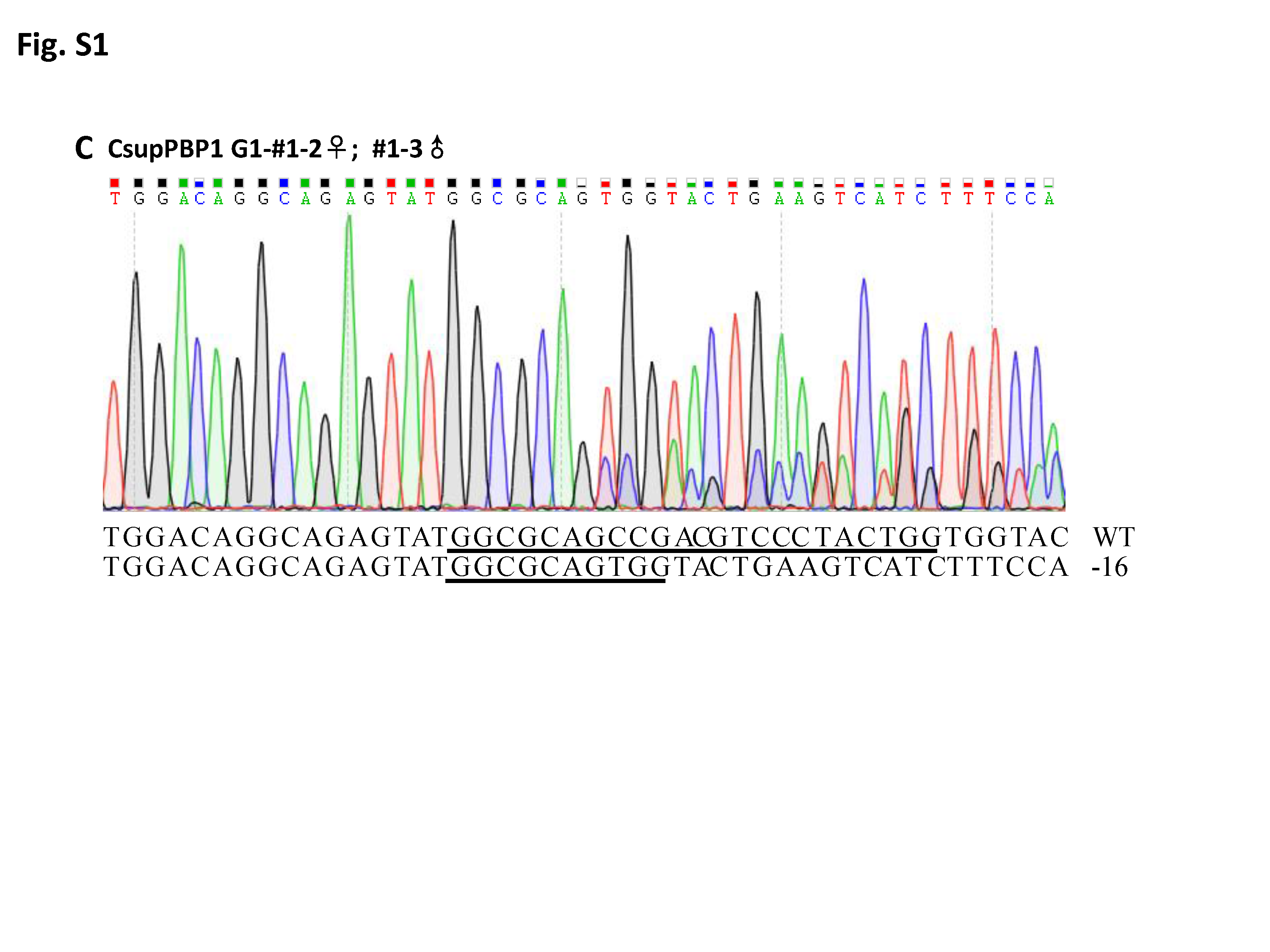

Supplement: Supplementary file 4 [file INS-26-388-s010.tiff]

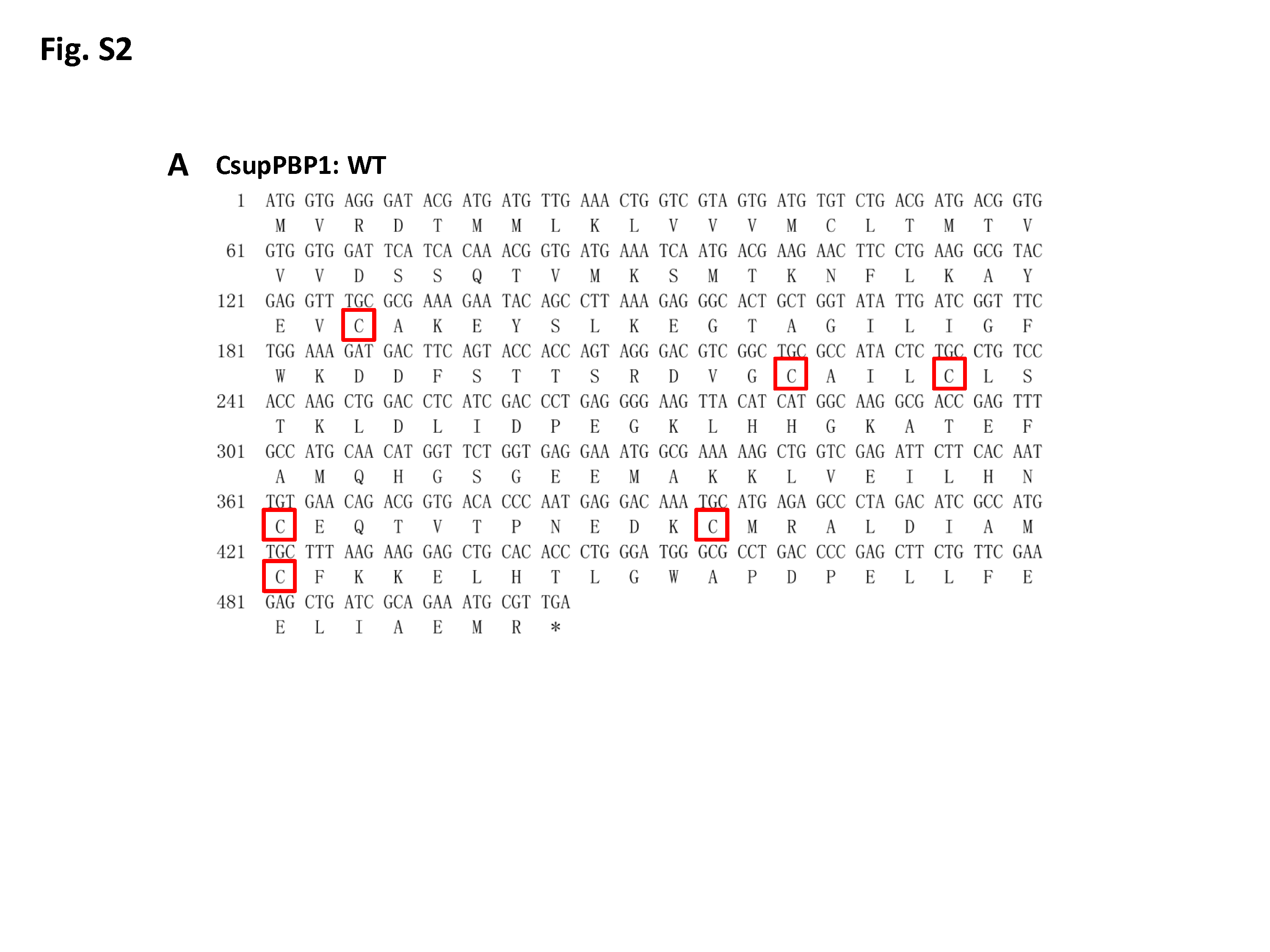

Supplement: Supplementary file 5 — Fig. S2 Amino acid sequence of PBP1 wild type (A) and G1 mutant (B and C) moths. The conserved cysteines (C) are boxed, showing six cysteines in the wild type sequence, and only one or two cysteines in the mutant sequences. [file INS-26-388-s011.tiff]

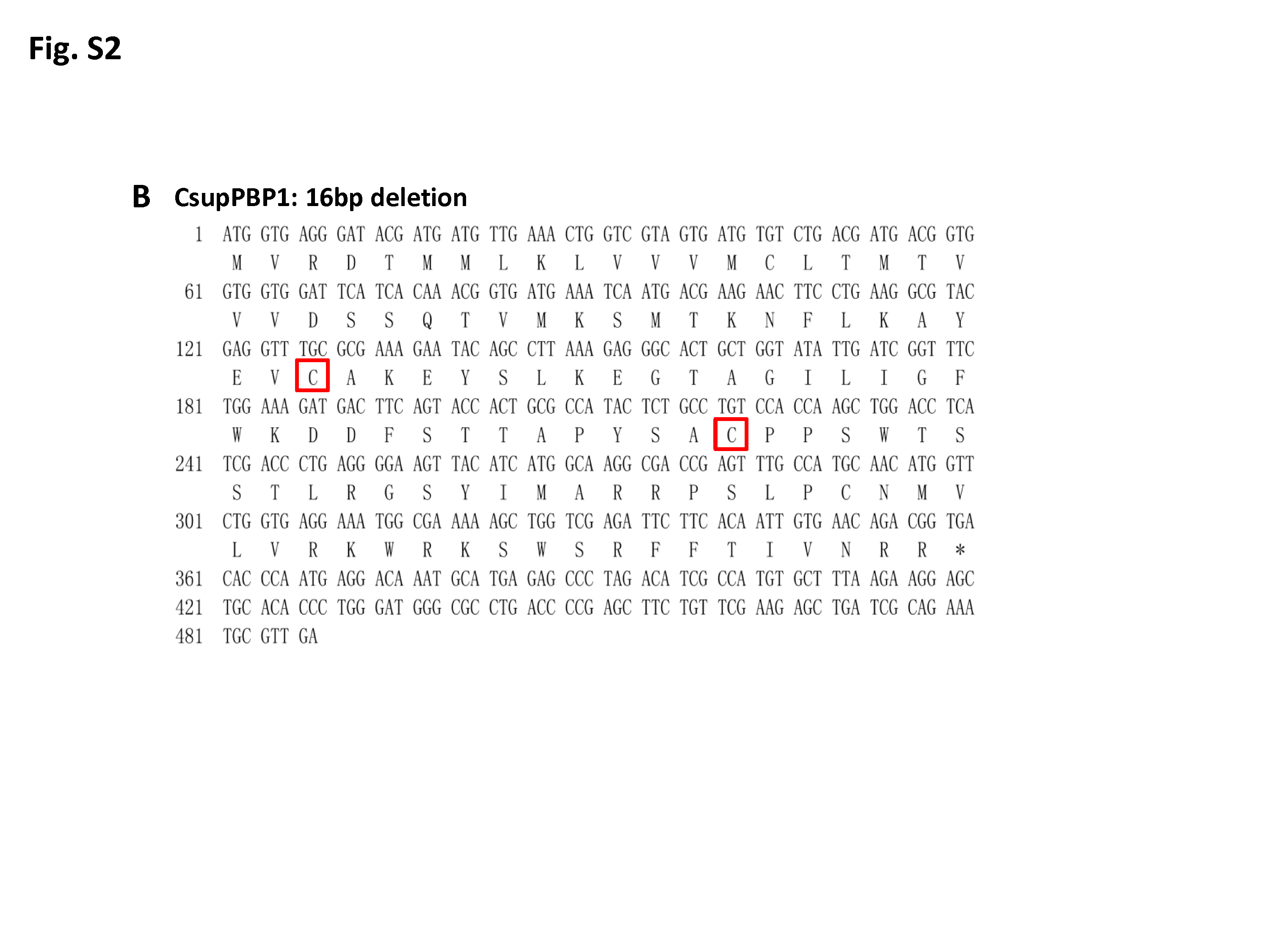

Supplement: Supplementary file 6 [file INS-26-388-s012.tiff]

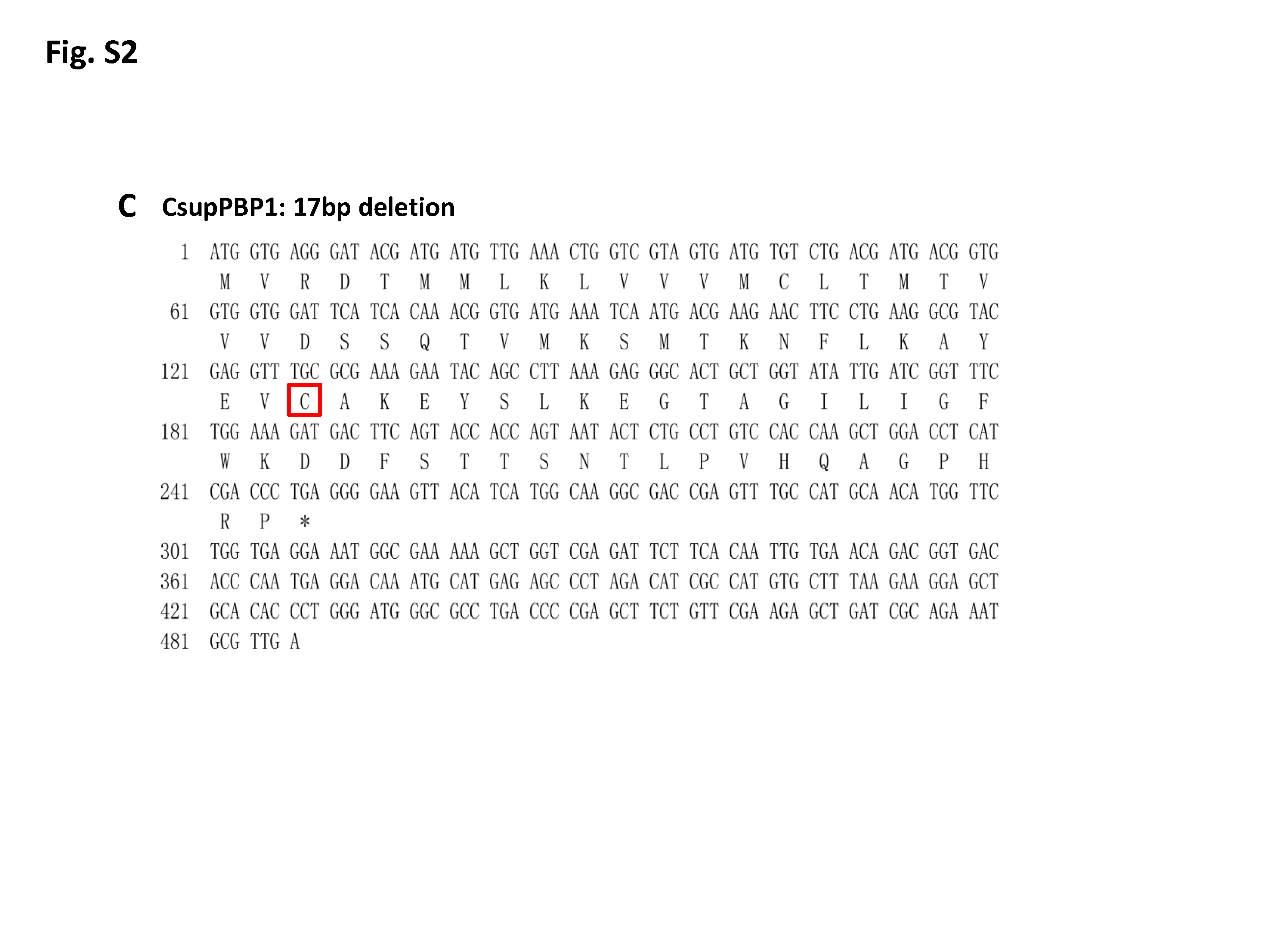

Supplement: Supplementary file 7 [file INS-26-388-s013.tiff]

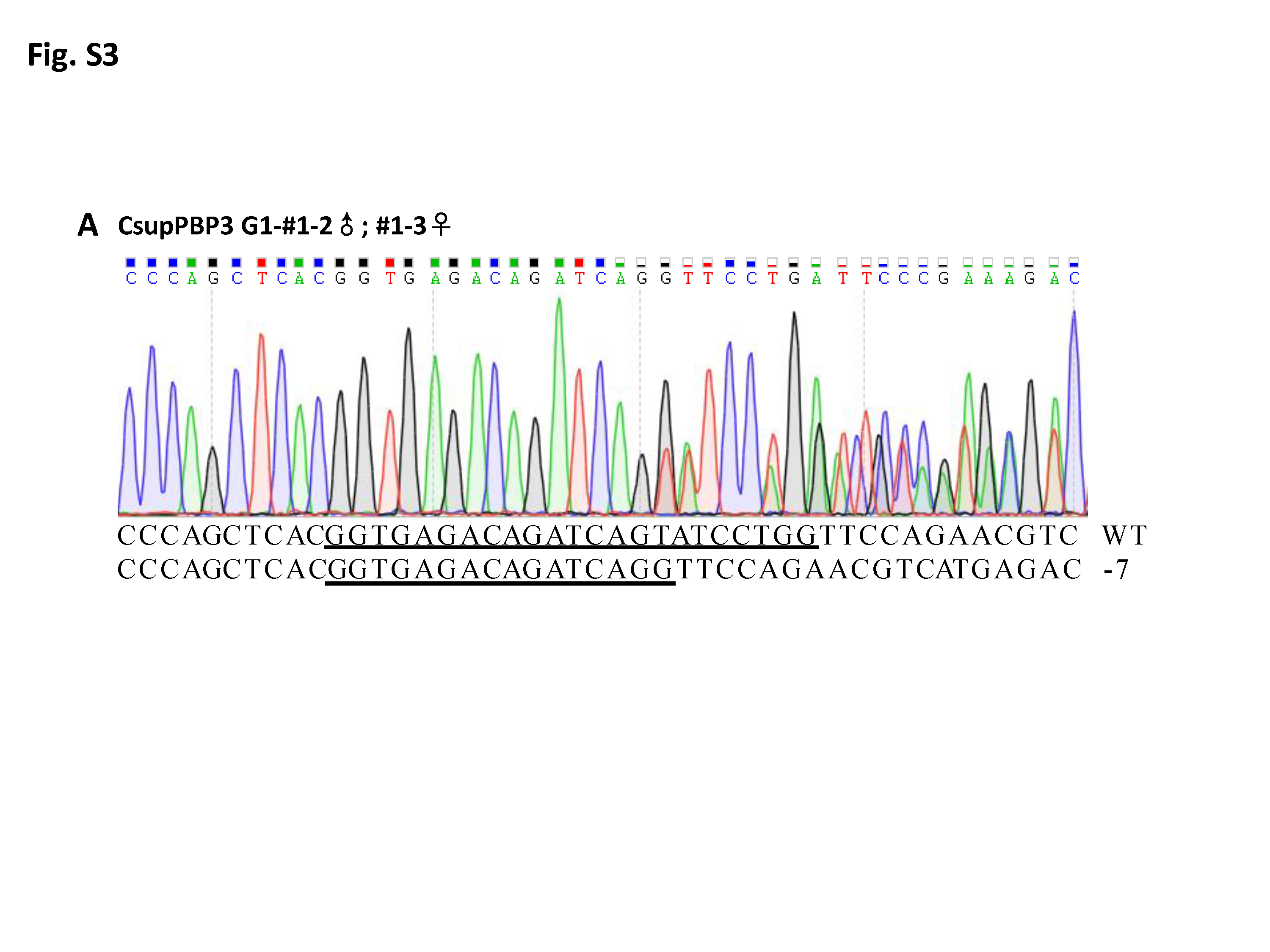

Supplement: Supplementary file 8 — Fig. S3 Representative chromatograms of PBP3 PCR products amplified by the gDNA from G1 moths that laid fertilized eggs. (A), (B) and (C) show PBP3 heterozygotes with 7 bp deletion, PBP3 homozygote with 12 bp deletion and PBP3 heterozygotes with 12 bp deletion, respectively. The stacked peaks indicate the heterozygotes, by direct sequencing of the PCR products; WT and “−7” or “−12” show the wild type and the mutant (a 7 bp or 12 bp deletion) sequences respectively, which are determined by TA cloning and sequencing. The target site is underlined. [file INS-26-388-s014.tiff]

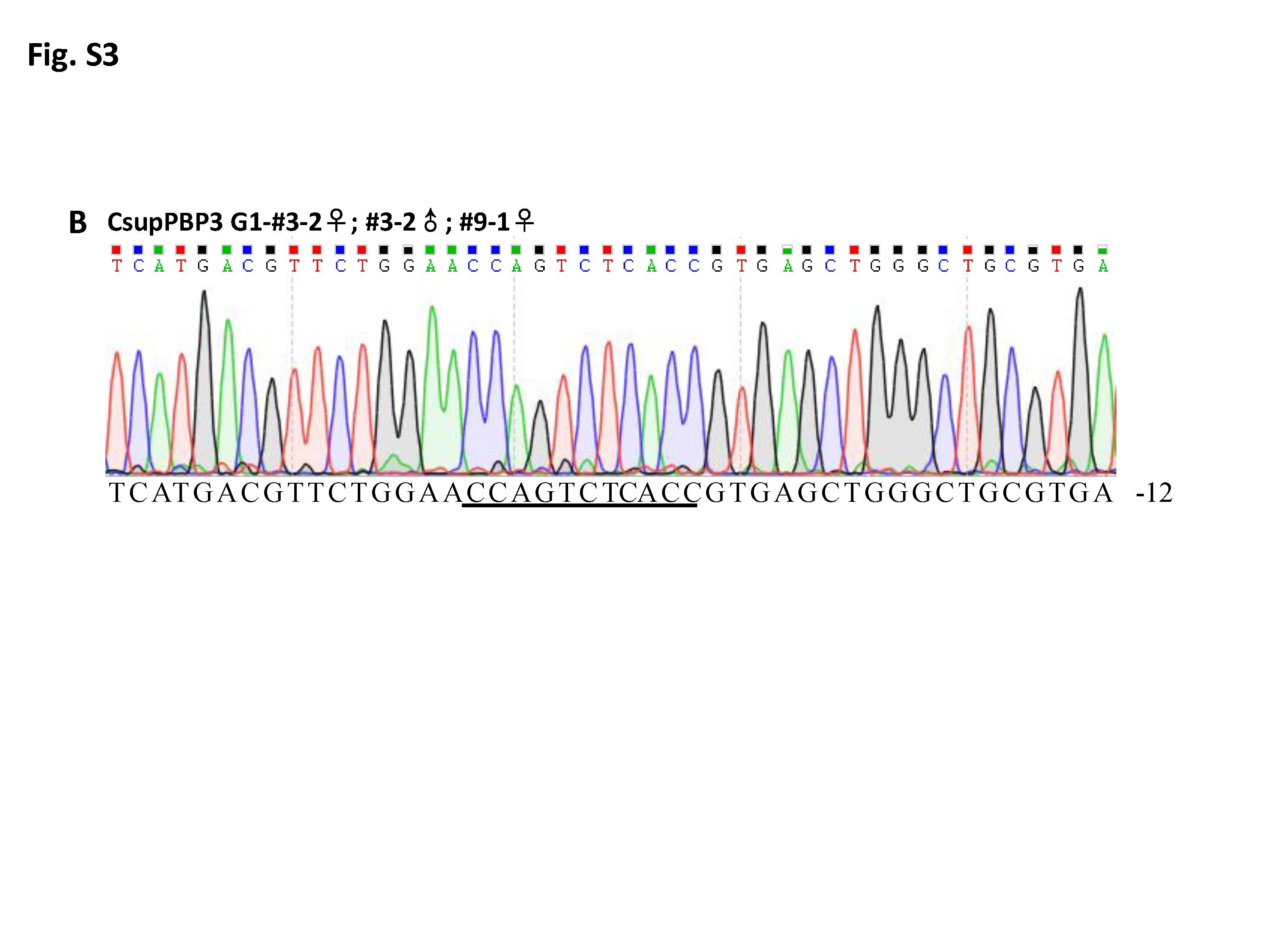

Supplement: Supplementary file 9 [file INS-26-388-s015.tiff]

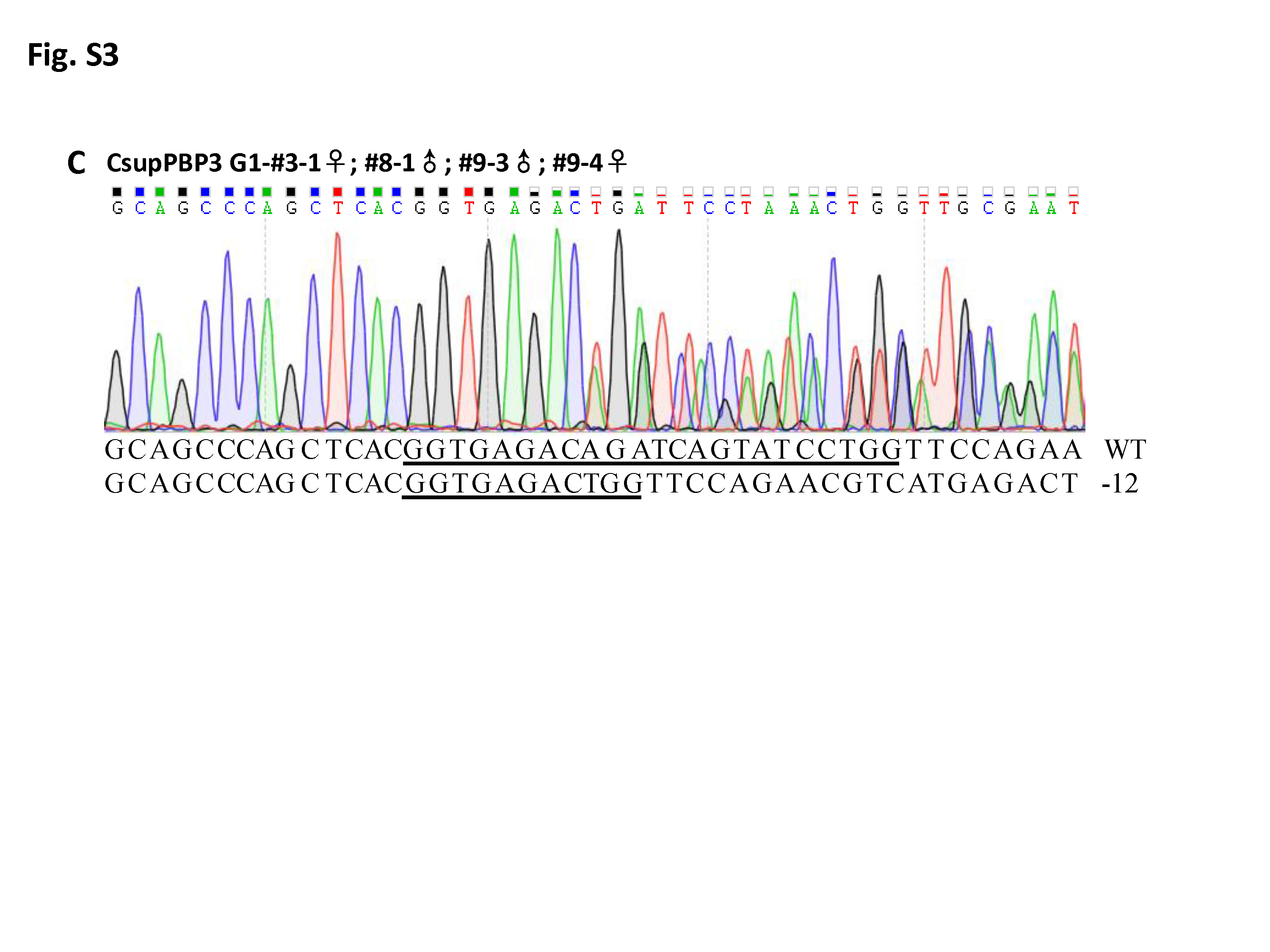

Supplement: Supplementary file 10 [file INS-26-388-s016.tiff]

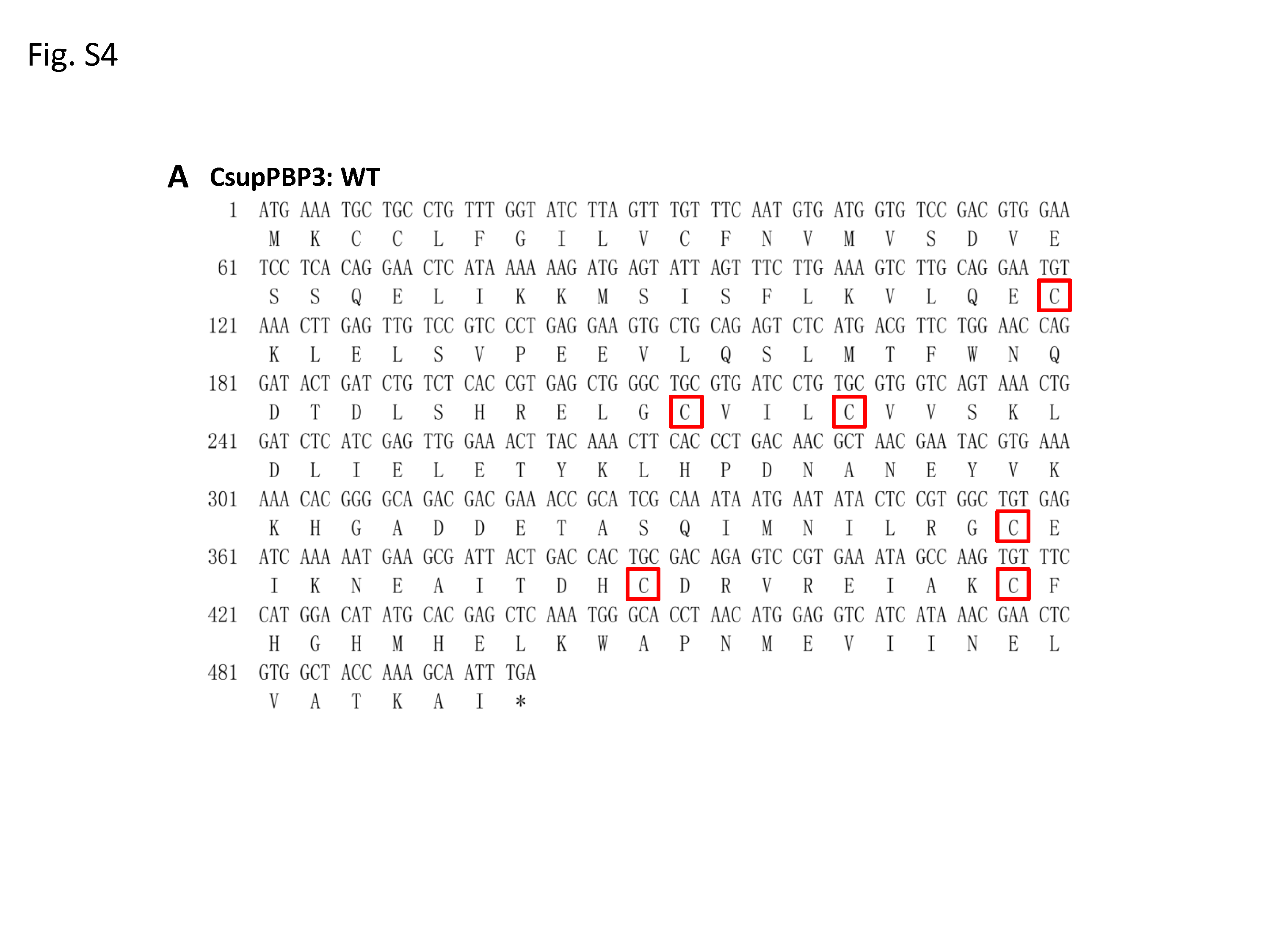

Supplement: Supplementary file 11 — Fig. S4 Amino acid sequence of PBP3 wild type (A) and G1 mutant (B and C) moths. The conserved cysteines (C) are boxed, showing six cysteines in the wild type sequence and the mutant sequence with 12 bp deletion, and only one cysteine in the mutant sequences with 7 bp deletion. [file INS-26-388-s001.tiff]

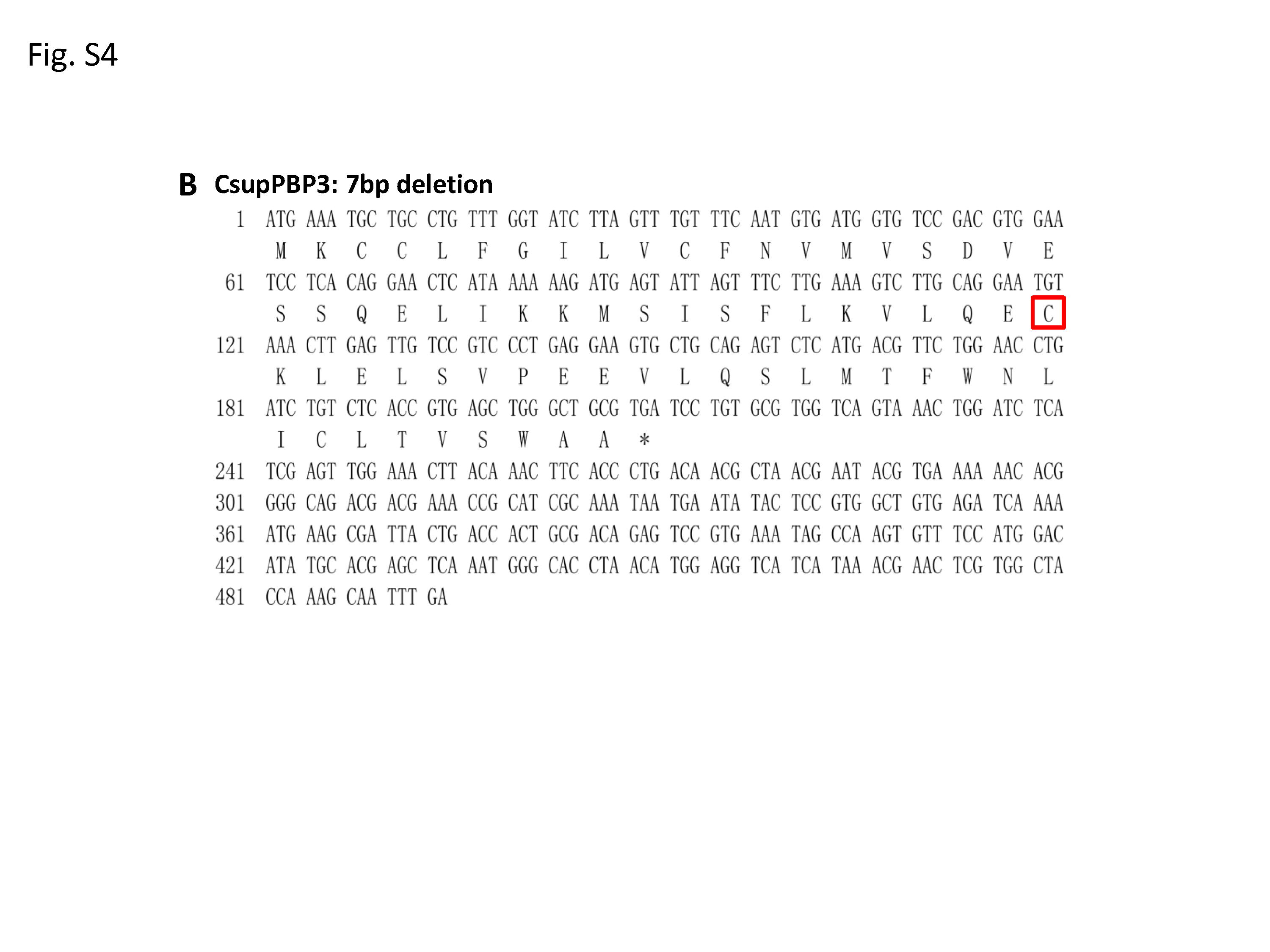

Supplement: Supplementary file 12 [file INS-26-388-s003.tiff]

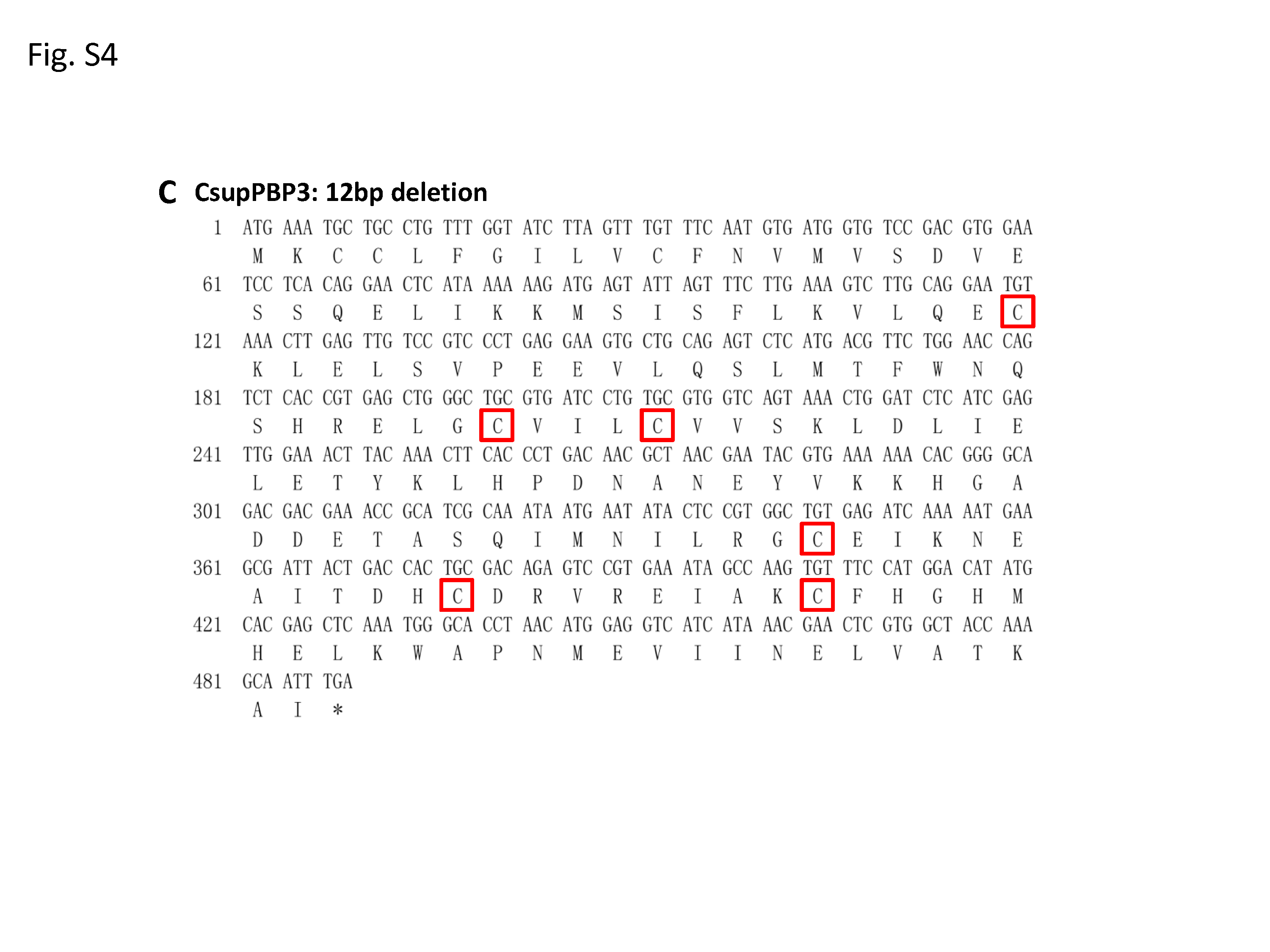

Supplement: Supplementary file 13 [file INS-26-388-s004.tiff]

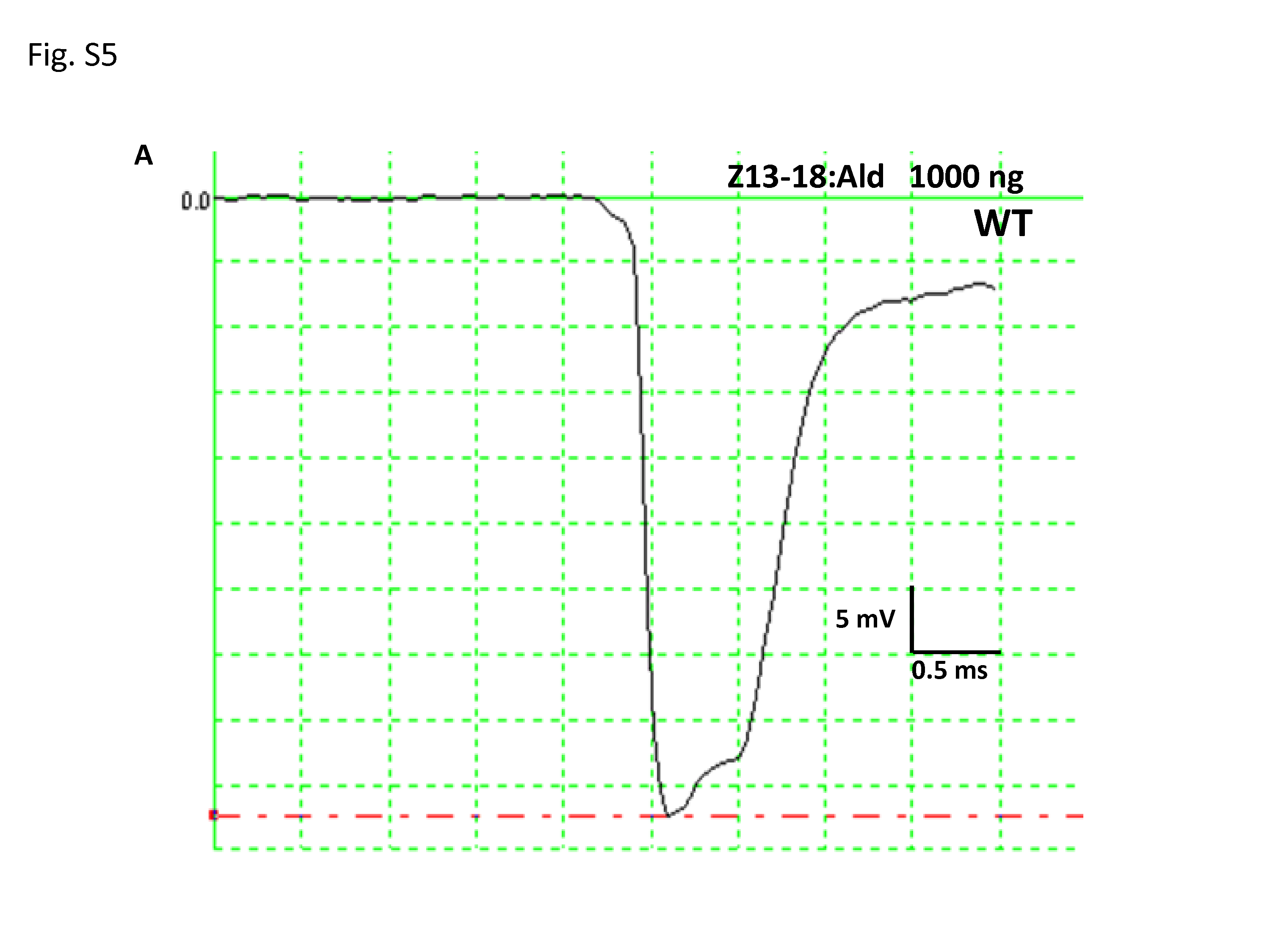

Supplement: Supplementary file 14 — Fig. S5 Typical EAG response diagrams of PBP1 wild type (A), PBP1+/− (B) and PBP1−/− (C) males to Z13–18:Ald (1000 ng). The EAG value for wild type, PBP1+/− and PBP1−/− males are 47.408, 30.436 and 26.589 mV, respectively. The scale of the schematics is 5 mV. [file INS-26-388-s005.tiff]

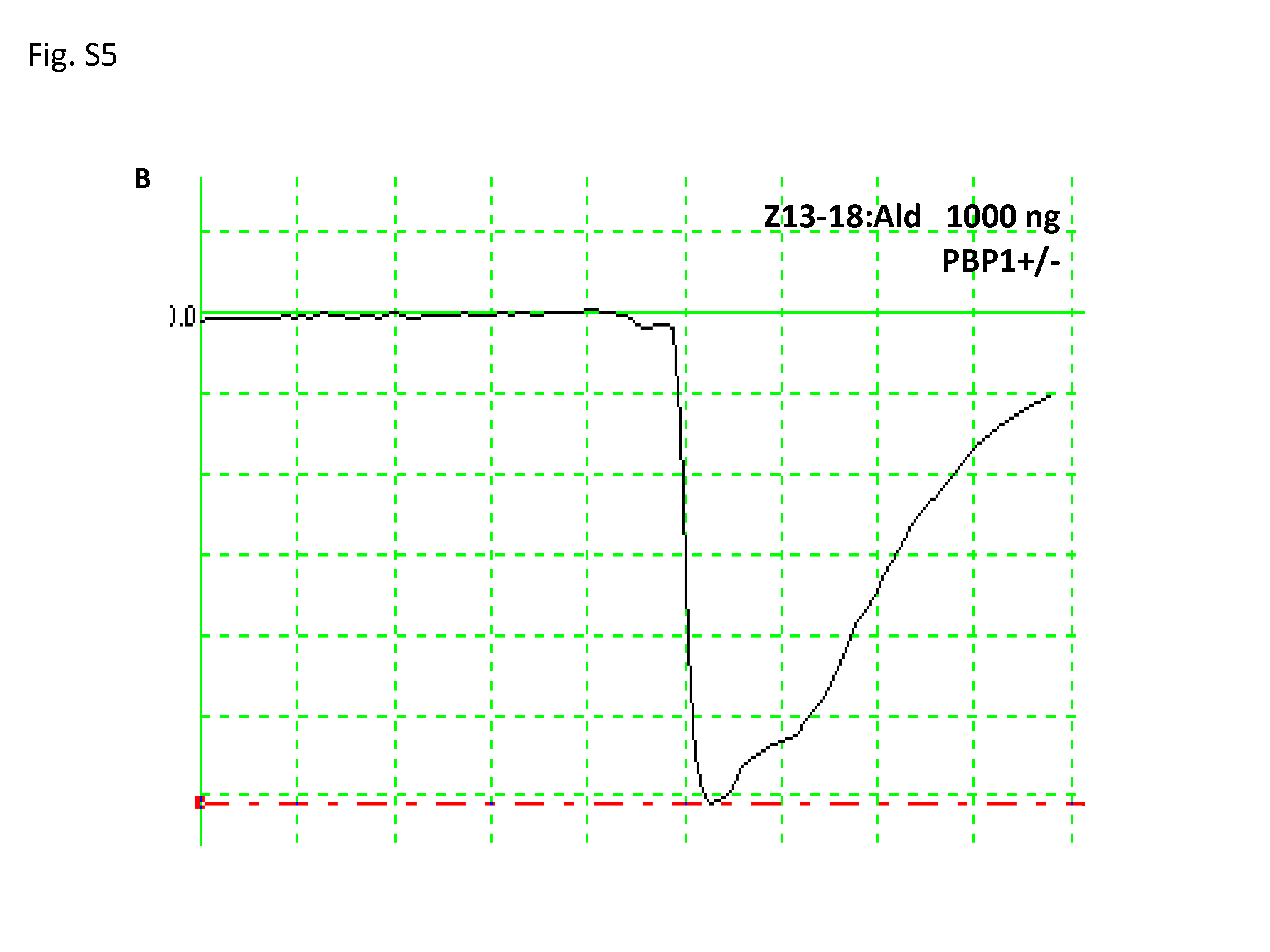

Supplement: Supplementary file 15 [file INS-26-388-s006.tiff]

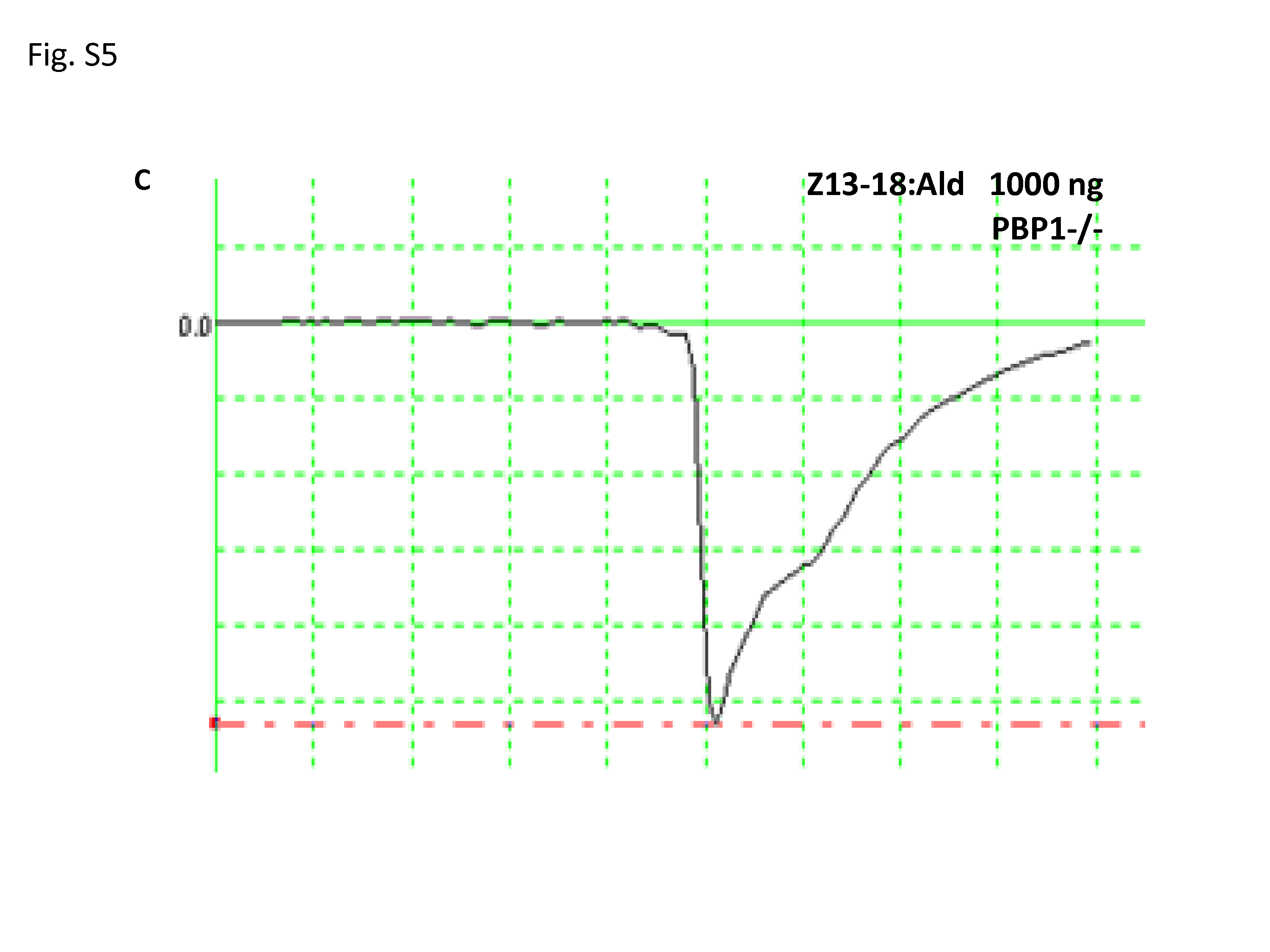

Supplement: Supplementary file 16 [file INS-26-388-s007.tiff]
